# One-pot synthesis, computational chemical study, molecular docking, biological study, and in silico prediction ADME/pharmacokinetics properties of 5-substituted 1*H*-tetrazole derivatives

**DOI:** 10.1038/s41598-023-44615-4

**Published:** 2023-10-19

**Authors:** Ahmed El-Sewedy, Eman A. El-Bordany, Naglaa F. H. Mahmoud, Kholoud A. Ali, Sayed K. Ramadan

**Affiliations:** 1 Chemistry Department, Faculty of Science, Ain Shams University, Cairo, 11566 Egypt; 2 Zoology Department, Faculty of Science, Ain Shams University, Cairo, 11566 Egypt

**Keywords:** Cancer, Chemical biology, Chemistry

## Abstract

An efficient synthesis of 5-substituted 1*H*-tetrazoles was successfully achieved through one-pot multi-component condensation reactions of some aromatic aldehydes or indolin-2,3-dione with malononitrile and sodium azide using diverse reaction conditions to obtain considerable product yields. Furthermore, it has been achieved for the first time to construct desired products under neat condition. Molecular docking studies with CSNK2A1 receptor disclosed the lowest binding energy displayed by the dimethoxyphenyl derivative **4c** with − 6.8687 kcal/mol. The synthesized tetrazoles were screened for their in-vitro cytotoxic activity against epidermoid cancer cell line (A431) and colon cancer line (HCT116) with respect to normal skin fibroblast cell line (BJ-1) using MTT assay, and antimicrobial activity against the bacteria: *K. pneumonia*, *S. aureus*, and the fungi: *Candida albicans*, as well as their antioxidant activity using 2,2-diphenyl-1-picrylhydrazyl assay. In addition, the toxicity of tetrazole derivative was assessed by determination of their approximate lethal dose fifty (LD_50_), calculated via an oral administration to rats, through measurement of ALT and bilirubin levels in serum. The antitumor results can suggest that the potent tetrazole derivative namely, *3-(3,4-dimethoxyphenyl)-2-(1H-tetrazol-5-yl)acrylonitrile ****(4c)*** could be a potential drug against epidermoid carcinoma. The antioxidant results indicated to tetrazoles exhibited great antioxidant properties even at very low doses. A molecular dynamics simulation was performed for the synthesized compounds (ligands) to investigate their tendency for binding with the active sites of protein.

## Introduction

Multi-component reactions (MCRs) combine three or more starting materials in a single chemical event to form a product containing most of the starting materials atoms. Mild conditions, efficiency, atom economy, straightforward reaction design, high affinity, and the progressive economy have been associated with modern multi-component reactions devices with their general compatibility with green chemistry^1–12^. Recently, the synthesis of tetrazole (conjugated nitrogen-rich heterocycles) scaffolds has suited outstanding in the field of chemical and pharmaceutical research due to their utility as a pharmacophore in many interesting fields. In 2019, it was reported by drug bank that 23 approved medicines contained tetrazole as its core moiety^13^.

**Figure 1:**
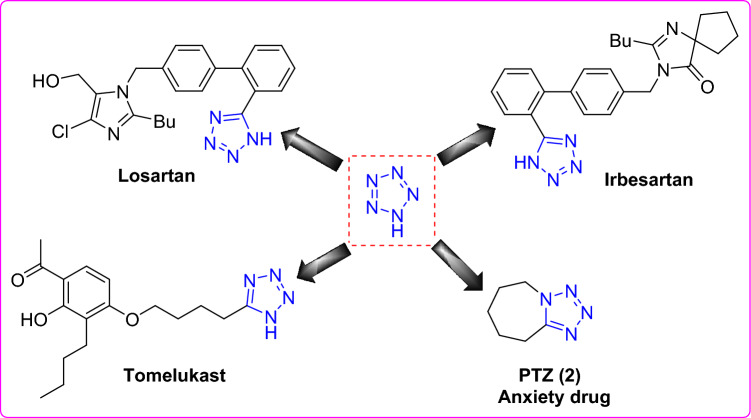
Representative tetrazoles-based drugs.

Therefore, tetrazole derivatives show a crucial role in pharmaceutical and medicinal applications^14,15^. Noteworthy, the tetrazole function is metabolically stable. Thus, this highlight and a close relationship between the acidic character of the tetrazole group and carboxylic acid group^16^ have encouraged medicinal chemists to prepare substituted tetrazoles as potential pharmacological agents (Fig. 1). Therefore, tetrazoles are considered as bio-isosteres of carboxylic acids. Molecular docking studies play a fundamental role in deciding design and synthesis of pharmacologically-relevant tetrazole derivatives.

Thus, design and synthesis of substituted tetrazoles have been approached in various methods like eco-friendly, non-toxic, water solvent, moderate conditions, easy setup, easy extractions, low cost, etc. with fair yields. A survey of literature revealed that 5-substituted 1*H*-tetrazole derivatives were synthesized through tandem [2 + 3] cyclo-addition reaction giving acrylates and coumarins including dimethyl formamide and ammonium chloride^17^. On the other side, it was developed via multi-component domino Knoevenagel condensation/1,3-dipolar cycloaddition reaction containing Fe_3_O_4_ magnetic nanoparticles using microwave irradiation. In the present research and in continuation to our strategy^18–28^, the one-pot multi-component reactions have been investigated to optimize the conditions for synthesis of 2-(1*H*-tetrazole-5-yl)acrylonitrile derivatives **4a–e** using some catalysts and various techniques to achieve facile, eco-friendly, and one-pot process through a domino Knoevenagel condensation and 1,3-dipolar cyclo-addition reaction.

## Results and discussion

### Chemistry

**Scheme 1:**
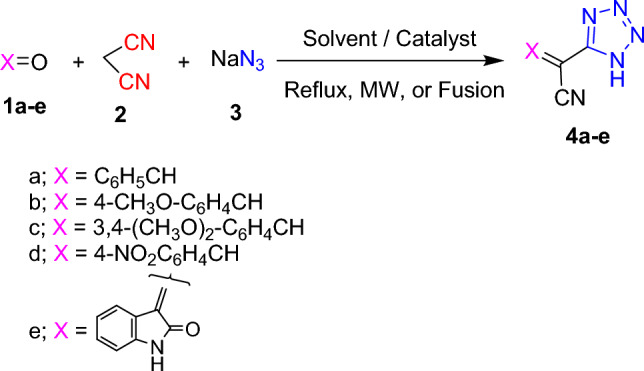
Synthesis of tetrazoles **4a–e** via MCR’s fashion.

The target 3-substituted-2-(1*H*-tetrazol-5-yl)acrylonitriles **4a–e** have been synthesized via preliminary investigation of one-pot multi-component reactions of different aromatic aldehydes like benzaldehyde, 4-methoxybenzaldehyde, 3,4-dimethoxybenzaldehyde, and 4-nitrobenzaldehyde **1a–d**, or the ketone, indoline-2,3-dione (**1e**) with malononitrile **2** and sodium azide **3** under different conditions (Scheme 1). The conventional technique included catalysts, solvents, and neat conditions. Noteworthy over the last years, the use of green chemistry tools like microwave and ultrasonic assisted techniques have developed as impressive methods for the synthesis of target molecules.

So, eco-friendly attempts for the synthesis of the target tetrazoles have been reported using microwave (MW) and ultrasonic (US) irradiation conditions. All structures of the synthesized compounds were based on their spectral and elemental analyses. Thus, their infrared spectroscopic analysis displayed the appearance of the absorption bands at ν 3390–3300, 3080–3030, and 2229–2219 cm^−1^ which were attributed to NH, Ar–H, and C≡N stretching frequencies, respectively. The IR spectrum of indolinone derivative **4e** displayed the nitrile and carbonyl functionalities at ν 2219 and 1714 cm^−1^, respectively, besides the two NH groups at ν 3375 and 3240 cm^−1^. Also, ^1^H NMR spectrum offered two exchangeable singlet signals at δ 10.84 ppm and δ 9.09 ppm corresponding to NH proton of indolinone ring and NH proton of tetrazole moiety, respectively.

The optimum reaction conditions (1 mmol of aldehydes, 1 mmol of malononitrile, and 1.5 mmol of sodium azide) were evaluated. First, solvents like ethanol and acetic acid under reflux were employed. In next attempt, some catalysts have been used like sodium ethoxide (EtONa), ceric ammonium nitrate (CAN), sodium acetate, and some Lewis acids like zinc chloride (ZnCl_2_), aluminum chloride (AlCl_3_), and ferric chloride (FeCl_3_) as a bulk materials and as a nanoparticles like ZnO, TiO_2_, NiFe_2_O_4_, CoFe_2_O_4_, and CuFe_2_O_4_. Also, eco-friendly techniques including microwave and ultrasonic irradiation were investigated. Last, the reaction was submitted to be investigated under fusion conditions. The yields, catalyst amounts, and reaction time were estimated. It was worthy that tetrazole derivatives **4a–e** were acquired in spectacular yields under fusion condition (solventless and catalyst-free) (cf. Table 1).

**Table 1:** Optimization of reaction conditions for synthesis of target tetrazole derivatives.

Entry	Catalyst/condition	Weight of catalyst (g)	Solvent	Temp. (°C)	Time (min.)	Yield %
1	–	–	Ethanol	Reflux	600	27
2	–	–	AcOH	Reflux	300	30
3	EtONa	–	Ethanol	Reflux	480	–
4	CAN	0.5	Ethanol	Reflux	480	–
5	ZnCl_2_	2	Ethanol	Reflux	720	–
6	AlCl_3_	2	Ethanol	Reflux	720	–
7	FeCl_3_	2	Ethanol	Reflux	900	–
8	AcONa	1.5	Ethanol	Reflux	300	40
9	ZnO	0.2	Ethanol	Reflux	360	60
10	TiO_2_	0.2	Ethanol	Reflux	300	65
11	NiFe_2_O_4_	0.2	Ethanol	Reflux	240	50
12	CuFe_2_O_4_	0.2	Ethanol	Reflux	300	55
13	CoFe_2_O_4_	0.2	Ethanol	Reflux	420	45
14	US	**–**	DMF	100	10	–
15	MW	**–**	DMF	100	4	62
16	Neat reaction	**–**	–	140	180	80

CAN: ceric ammonium nitrate; AcONa: sodium acetate; AcOH: acetic acid.

**Scheme 2:**
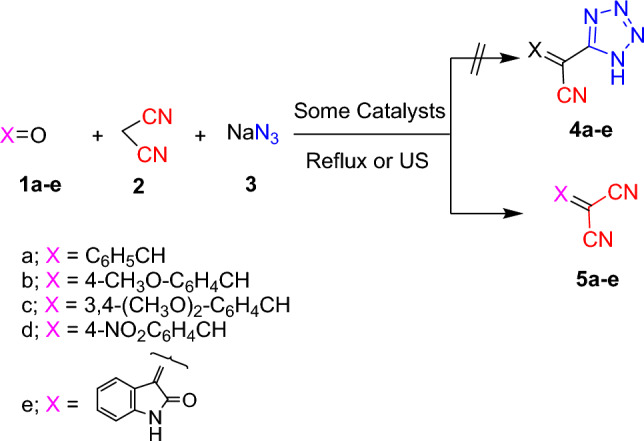
Using some bulk material catalysts and ultrasonic irradiation conditions.

The comparative results in Table 1 disclosed that using ethanol and acetic acid solvents under reflux condition afforded the target compounds in low yields. While, using the catalysts; sodium ethoxide, ceric ammonium nitrate, zinc chloride, aluminum chloride, and ferric chloride did not afford the desired products and furnished instead the arylidine malononitrile derivatives **5a–e** (Scheme 2). Otherwise, using sodium acetate as a catalyst afforded the desired product with 40% yield. It is worth mentioning that zinc oxide is a non-toxic, environmentally friendly, inexpensive, and non-hygroscopic white powder. In addition, it holds suitable characteristics as being safe and easily available. Also, it acts as Lewis acid catalyst which has gained much interest in many organic transformations.

Therefore, using ZnO, TiO_2_, NiFe_2_O_4_, CoFe_2_O_4_, and CuFe_2_O_4_ nanoparticles as catalysts enhanced the reaction yields (Entries 9–13). Otherwise, execution of the reaction under microwave irradiation conditions increased the reaction yield. Whereas, applying the ultrasonic irradiation conditions failed to give the desired products and afforded instead the arylidine malononitrile derivatives **5a–e** (Scheme 2). As a premier trial, spectacular yields of 2-(1*H*-tetrazol-5-yl)acrylonitrile derivatives **4a–e** have been successfully achieved upon carrying out the multi-component reaction under fusion condition, as a noticeable style toward environmentally clean synthesis of organic compounds.

After reaching our optimal values, we subsequently studied the substrate scope for our synthetic protocol. Therefore, four substituted aromatic aldehydes **1a–d** or indoline-2,3-dione **1e** were treated with malononitrile **2** and sodium azide **3**. Aldehydes with electron-withdrawing group (4-NO_2_) gave the desired product with excellent yield. Afterwards, aldehydes with electron donating groups (4-OCH_3_, 3,4-(OCH_3_)_2_) afforded the desired products with relatively lower yields.

**Scheme 3:**
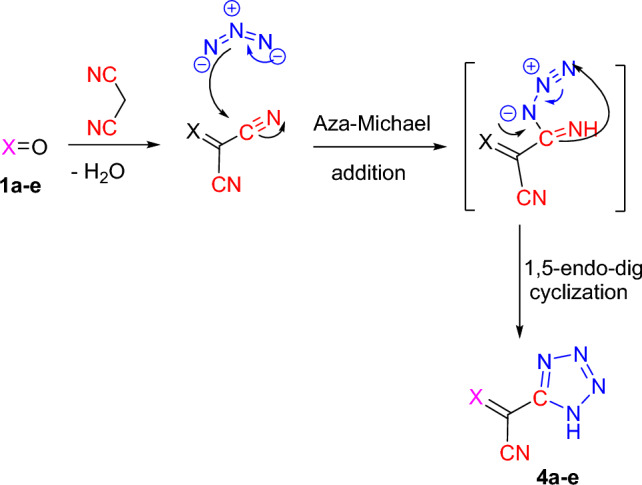
A plausible pathway for the formation of tetrazoles **4a–e**.

The formation of coveted products might be elucidated through the formation of arylidene malononitrile followed by aza-Michael addition of azide nitrogen ion on nitrile carbon atom of arylidene moiety forming the intermediate which underwent 1,5-endo-dig cyclization to form tetrazole as a final product (cf. Scheme 3). It seemed interesting that the presence and the nature of substituents on the aldehyde greatly affect the reactivity and outcoming yields. Like, when the following electron-withdrawing moiety such as -NO_2_ was introduced on the aldehydes, it raised the electron deficiency of the electrophilic center on aldehydic group which facilitated the nucleophilic attacking on it by the active methylene of malononitrile. In the meantime, the electron-donating groups such as –OCH_3_, and 3,4-(OCH_3_)_2_ moieties have counter effects.

**Figure 2:**
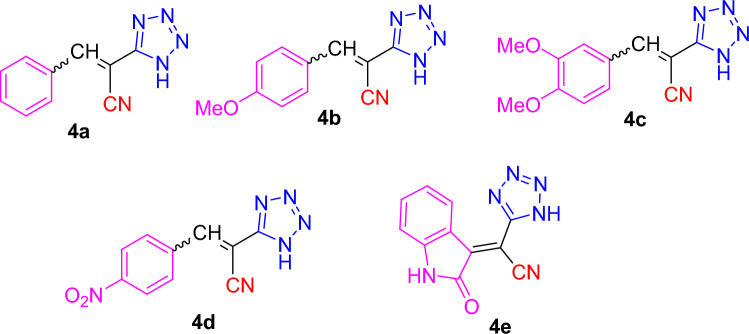
Structures of 2-(1*H*-tetrazole-5-yl)acrylonitrile derivatives **4a–e** obtained.

As our inference, the optimized synthetic methodology of 2-(1*H*-tetrazol-5-yl)acrylonitrile derivatives was achieved by solvent-free one-pot multi-component reactions in the absence of catalyst with spectacular yields. Therefore, this fashion enables to construct a wide variety of 2-(1*H*-tetrazole-5-yl) acrylonitrile derivatives (cf. Fig. 2).

### Biology

#### Molecular docking

Docking Score is the scoring function used to predict the binding affinity of both ligand and target once it is docked. The negative result indicates that the complex is more stable. This means that a very negative score corresponds to a strong binding and a less negative or even positive score corresponds to a weak or non-existing binding score. The score is listed in the docking results table as "Score".

**Figure 3:**
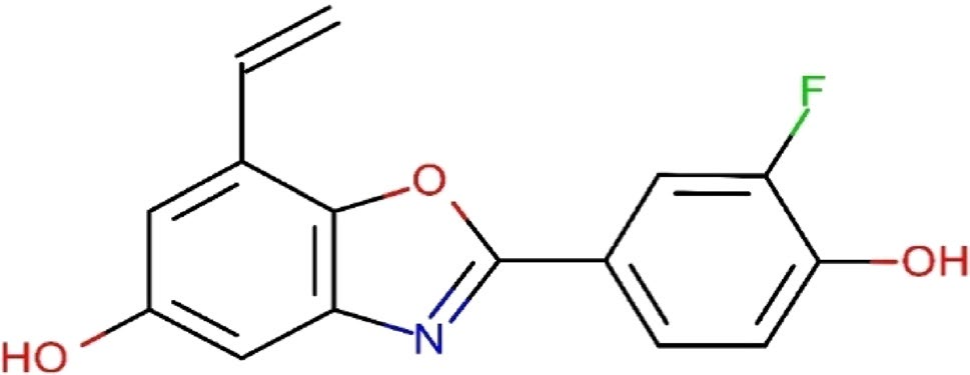
Structure of the reference ligand, ERB-041.

**Figure 4:**
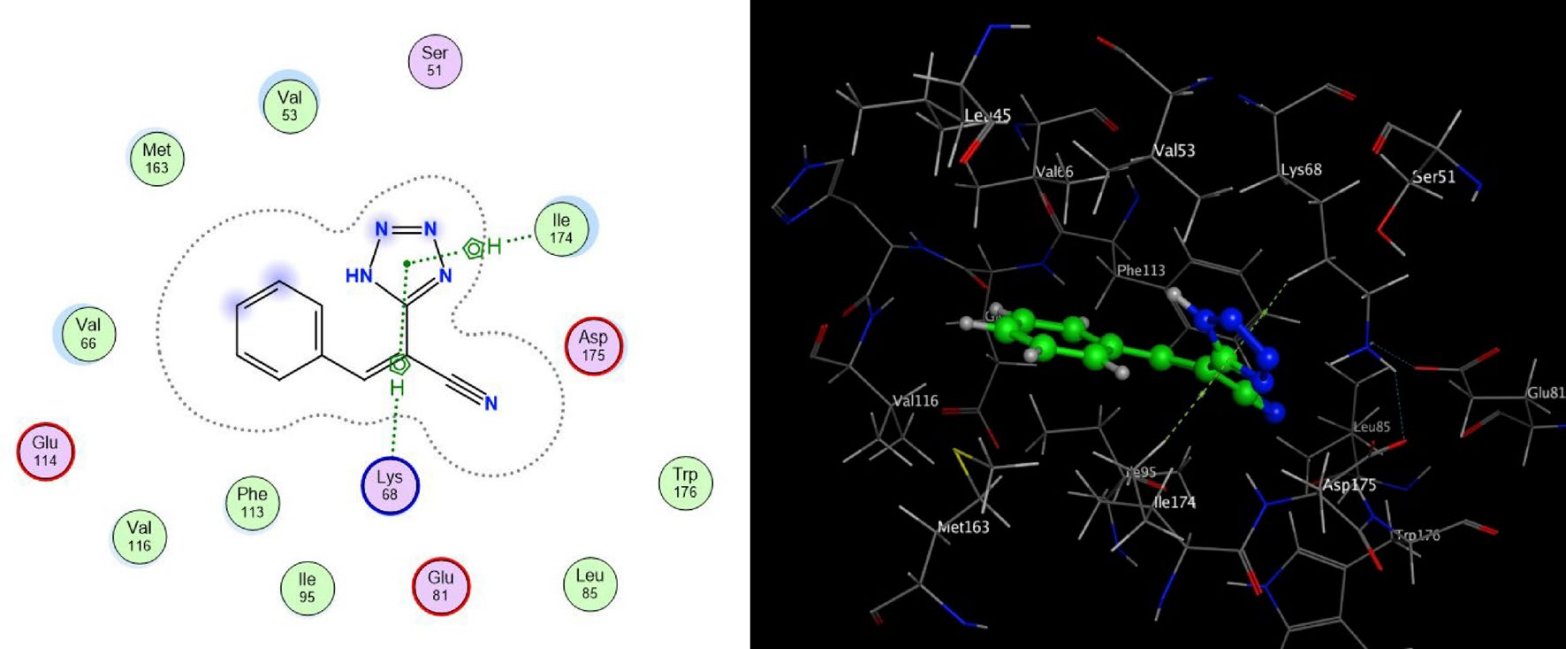
Proposed binding modes of compound **4a** docked in the active site of CSNK2A1 enzyme; 2D (Left) and 3D (Right) ligand-receptor interactions (hydrogen bonds are illustrated as arrows; C atoms are colored gray, N blue, and O red).

For a great understanding of the intermolecular interactions, molecular docking studies were carried out between all the synthesized ligands and CSNK2A1 receptors. The molecular docking study has been executed on the synthesized tetrazole derivatives as unprecedented ligand in inhibition of CSNK2A1 which was restored from protein data bank (PDB: 6QY7) (http://www.rcsb.org/pdb) compared to the ligand 2-(3-fluoro-4-hydroxyphenyl)-7-vinylbenzo[*d*]oxazol-5-ol (ERB-041) (Fig. 3) as a reference ligand for docking score. The molecular docking results can be displayed in Table 2. The docking score detected that compound **4c** showed the highest binding to CSNK2A1 enzyme with binding energy of − 6.8687 kcal/mol, this pointing to side chain donor of oxygen of the ligand and LYS 68 of enzyme in addition to other type of interaction called ligand exposure at four sites methyl group, nitrogen in tetrazole ring, nitrogen of cyano group and CH as shown in Fig. 4.

**Table 2:** The molecular docking scores of the investigated compounds **4a–e**.

Compd.	Docking score (kcal/mol)	Interaction	Distance	E (kcal/mol)
**ERB-041**	− 6.4434			
**4a**	− 5.9949	pi-H LYS 68	3.59	− 0.7
		pi-H ILE 174	4.06	− 0.9
**4b**	− 6.6210	H-donor MET 163	3.69	− 0.5
		H-acceptor ASP 175	3.25	− 0.5
		H-acceptor LYS 68	3.1	− 0.5
		H-pi PHE 113	3.59	− 0.5
		pi-H ILE 174	3.86	− 1.6
**4c**	− 6.8687	H-acceptor LYS 68	2.91	− 3.8
**4d**	− 6.4305	H-acceptor TRP 176	3.74	− 0.8
		pi-H ILE 174	4.15	− 0.7
**4e**	− 6.131	H-acceptor LYS 68	3.43	− 0.9
		pi-H VAL 53	3.8	− 0.8

While the compound **4b** showed binding to CSNK2A1 enzyme (with binding energy of − 6.6210 kcal/mol) higher than the reference ligand 041 with binding energy of − 6.4434 kcal/mol, this could be attributed to arene-H interaction between tetrazole ring of the ligand and ILE 174 of the enzyme and the ligand exposure. While compound **4d** has analogous inhibitory activity compared to reference ligand which have a binding energies − 6.4305 kcal/mol. Hence, the high active participation of the tetrazole rings and various substituents of these compounds was found to be very important for locking their geometries in the active site of the CSNK2A1 receptor.

**Figure 5:**
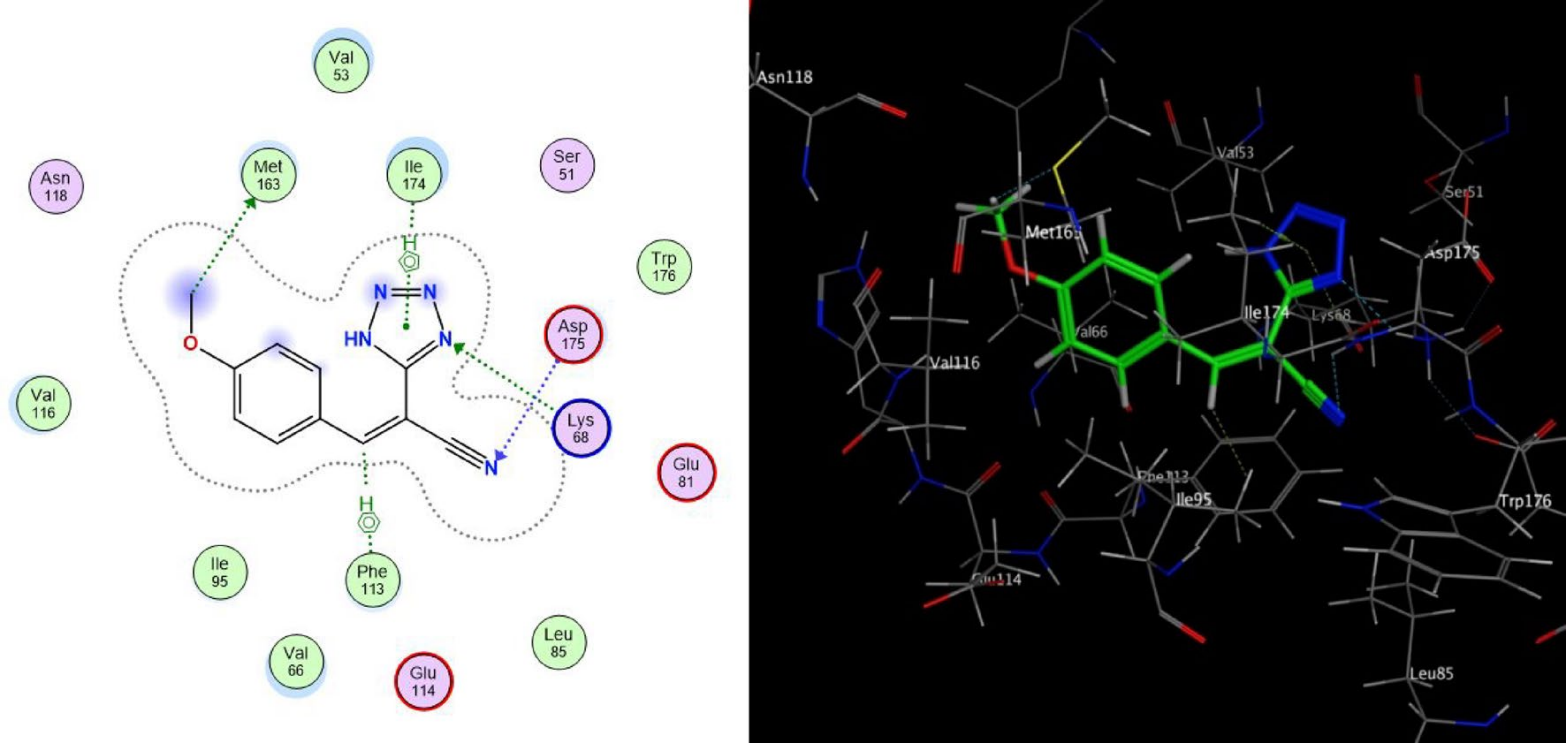
Proposed binding modes of compound **4b** docked in the active site of CSNK2A1 enzyme; 2D (Left) and 3D (Right) ligand-receptor interactions (hydrogen bonds are illustrated as arrows; C atoms are colored gray, N blue, and O red).

**Figure 6:**
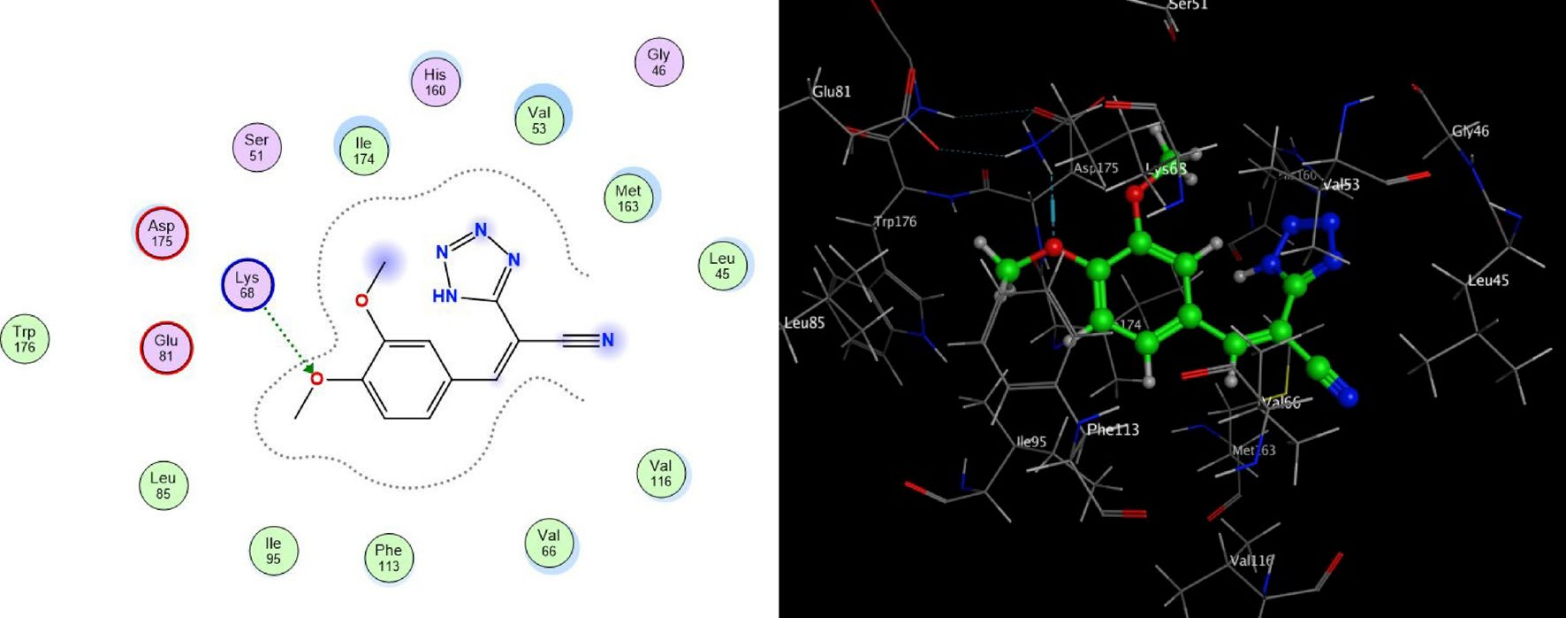
Proposed binding modes of compound **4c** docked in the active site of CSNK2A1 enzyme; 2D (Left) and 3D (Right) ligand-receptor interactions (hydrogen bonds are illustrated as arrows; C atoms are colored gray, N blue, and O red).

**Figure 7:**
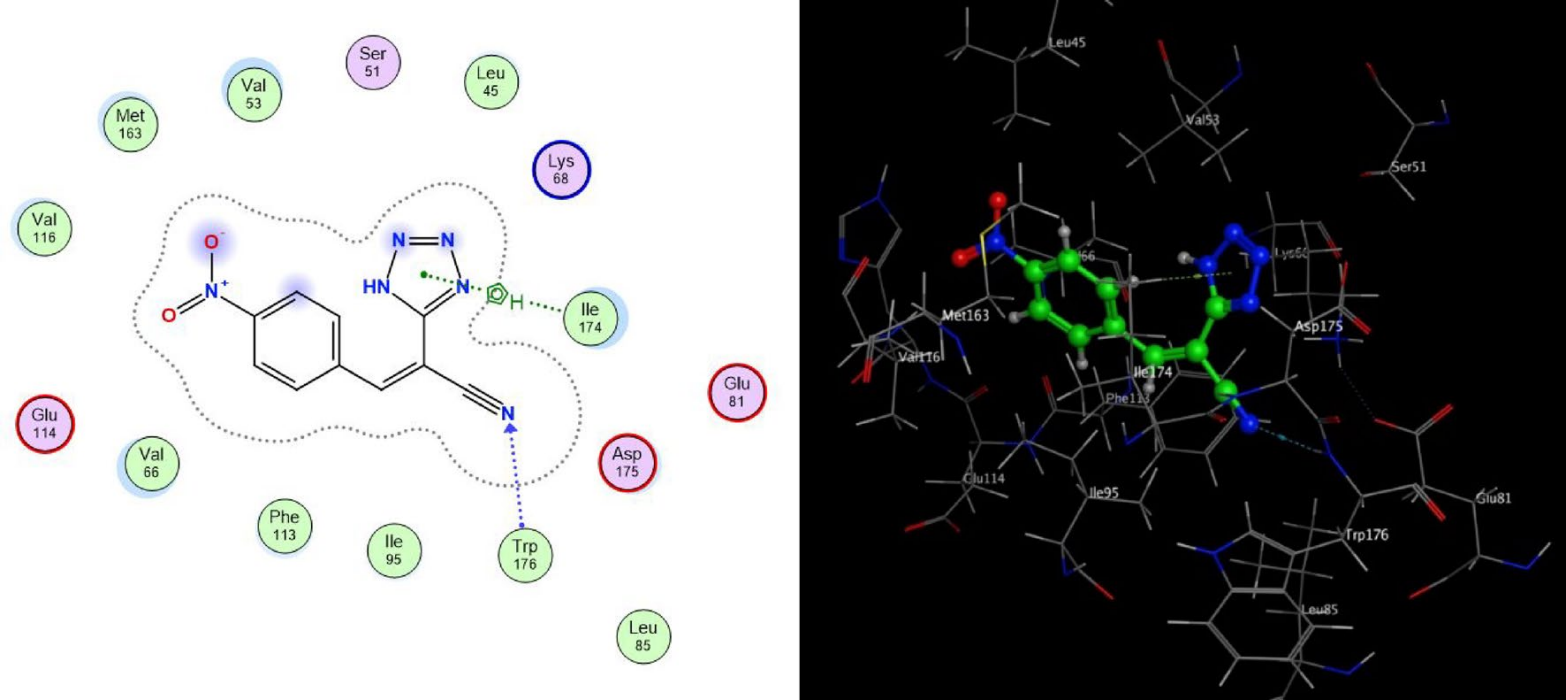
Proposed binding modes of compound **4d** docked in the active site of CSNK2A1 enzyme; 2D (Left) and 3D (Right) ligand-receptor interactions (hydrogen bonds are illustrated as arrows; C atoms are colored gray, N blue, and O red).

**Figure 8:**
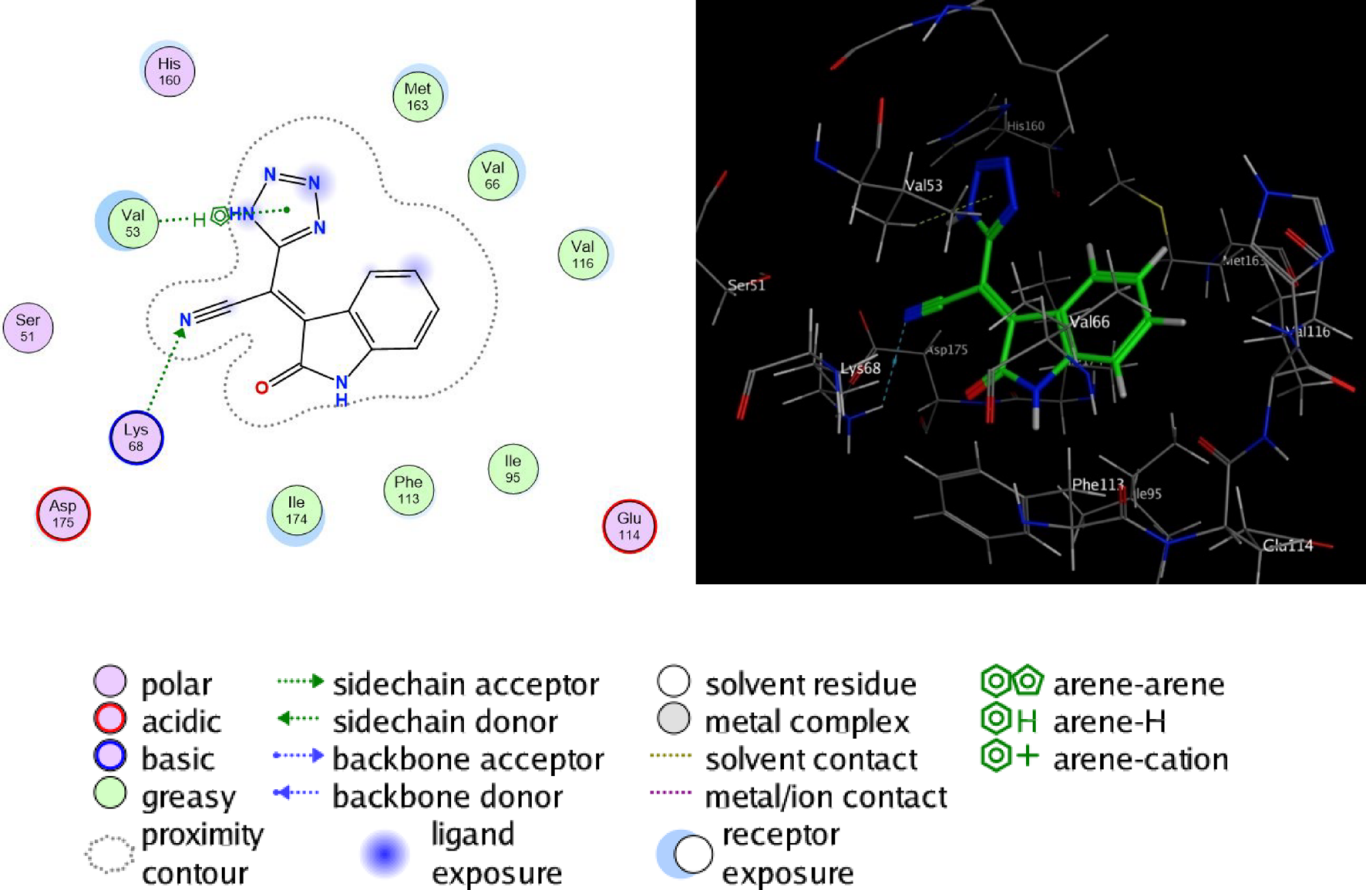
Proposed binding modes of compound **4e** docked in the active site of CSNK2A1 enzyme; 2D (Left) and 3D (Right) ligand-receptor interactions (hydrogen bonds are illustrated as arrows; C atoms are colored gray, N blue, and O red).

The interaction between the tetrazole derivatives **4a–e** and the active sites of CSNK2A1 enzyme were showed in 2D and 3D diagrams (cf. Figs. 4, 5, 6, 7, 8).

#### Cytotoxic activity evaluation

**Figure 9:**
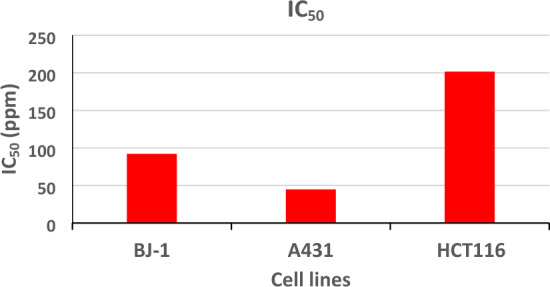
IC_50_ values of the tested compound **4c** as antitumor agent.

The anticancer effect of the tested tetrazole derivative **4c** was estimated using MTT assay^29^ against epidermoid carcinoma (A431) and colon cancer cell line (HCT116), as well as the normal skin fibroblast cells (BJ-1) at concentrations of 12.5, 25, 50, and 100 µg/mL. The well-known anticancer agent doxorubicin was used as a positive control. The results in Table 3 and Fig. 9 revealed that the highest IC_50_ values were found against HCT116 (colon cancer) to reach 201.45 ppm. While 92.05 ppm for BJ-1 (normal skin cells), and the lowest values were found against A431 at 44.77 ppm (Fig. 9, cf. Supporting information). These results can suggest that this potent compound could be a potential drug against epidermoid carcinoma.

**Table 3:** The IC_50_ values of the tested compound **4c** as antitumor agent.

Cell line	BJ-1				A431				HCT116			
Concn	12.5	25	50	100	12.5	25	50	100	12.5	25	50	100
Antitumor effect	6.836	37.543	39.356	48.666	10.646	54.8	62.886	75.133	0	0	10.4	20.9
IC_50_ (µg/mL)	92.045				44.77				201.45			

Results are expressed as mean ± S.E.M., n = 3, With *P* < 0.05 level of significance.

### Antimicrobial activity screening

#### Minimal inhibitory concentration (MIC)

**Figure 10:**
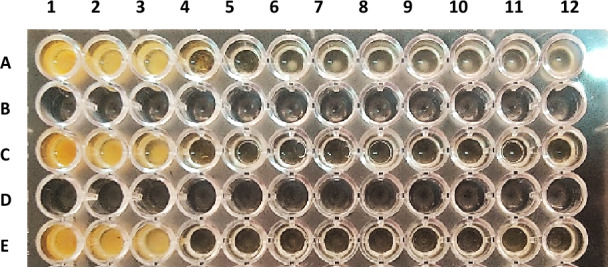
Antimicrobial effect of compound **4a** on (A) *K. pneumonia,* (C) *S. aureus,* (E) *C. albicans*.

**Figure 11:**
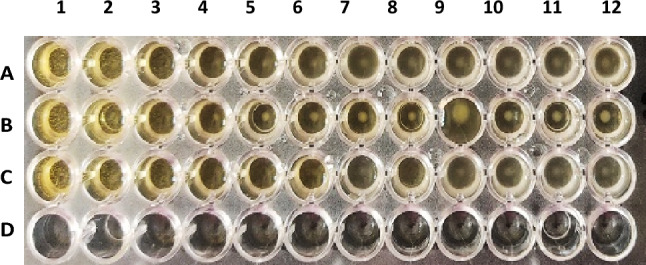
Antimicrobial effect of compound **4c** on (A) *K. pneumonia,* (B) *S. aureus,* (C) *C. albicans*.

**Figure 12:**
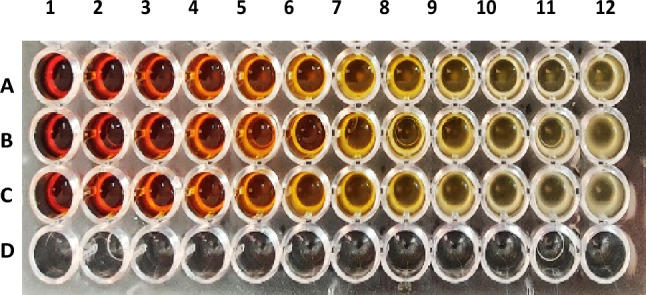
Antimicrobial effect of compound **4e** on (A) *K. pneumonia,* (B) *S. aureus,* (C) *C. albicans*.

The tetrazole derivatives **4a, 4c,** and **4e** were screened for their antibacterial activity against *K. pneumonia* (as Gram negative bacteria) and *S. aureus* (as Gram positive bacteria), and antifungal activity against *Candida albicans*. The results were displayed in Table 4 and Figs. 10, 11, 12. For antibacterial activity, compound 4a was the most active against *K. pneumonia* and *S. aureus* at concentrations as low as 6.25 and 1.56 mg/mL, respectively. Compounds **4e** and **4c** showed relatively higher MICs values against *K. pneumonia* (25 and 50 mg/mL, respectively) and *S. aureus* (50 mg/mL). All the tested compounds displayed weak antifungal activity (100 mg/mL), except compound **4c** which disclosed good activity against *C. albicans* at 12.5 mg/mL.

**Table 4:** The Minimal inhibitory concentrations (MIC, mg/mL) of compounds **4a**, **4c**, and **4e**.

Compd.	Strain (MIC, mg/mL)		
	*Klebsiella pneumonia*	*Staphylococcus aureus*	*Candida albicans*
**4a**	6.25	1.56	100
**4c**	25	50	12.5
**4e**	50	50	100

### Antioxidant activity screening

#### DPPH radical scavenging activity

**Figure 13:**
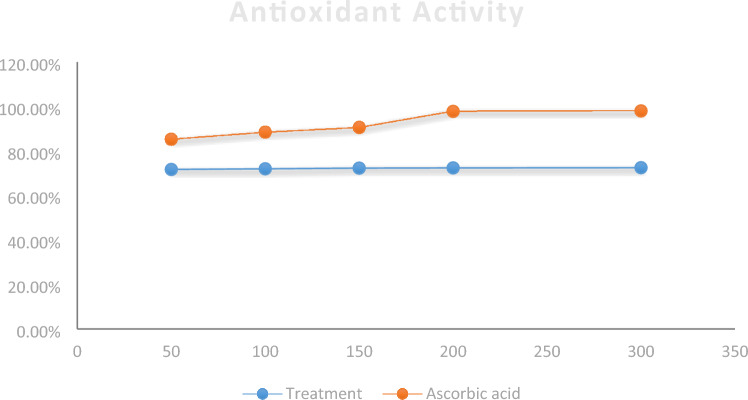
Radical scavenging activities of the tested compound compared to ascorbic acid.

The tetrazole derivative **4c** was tested for its antioxidant activity using the 2,2-diphenyl-1-picrylhydrazyl (DPPH) assay^30^. The radical scavenging activity of this tetrazole derivative was almost the same along the different concentrations against DPPH⋅. Ranging from 71.7% at 50 mg/mL to 72.5% at 300 mg/mL. DPPH⋅ scavenging activity of the compound against ascorbic acid is depicted in Table 5 and Fig. 13. The results showed that although ascorbic acid is superior to the tetrazole yet, the compound displayed a dose-independent radical scavenging activity. With increasing the dose many folds the compound antioxidant capacity remains more or less the same. Which means it has great antioxidant properties even at very low doses.

**Table 5:** Radical scavenging activity of the tested compound **4c** compared to ascorbic acid*.

Concentration mg/mL	Sample radical scavenging activity (%)	Ascorbic acid radical scavenging activity (%)
50	71.7	85.4
100	72	88.5
150	72.3	90.6
200	72.4	97.8
300	72.5	98

*T test was applied with *P* < 0.05 level of significance.

### Lethal dose fifty (LD_50_) calculation and body weight change

**Figure 14:**
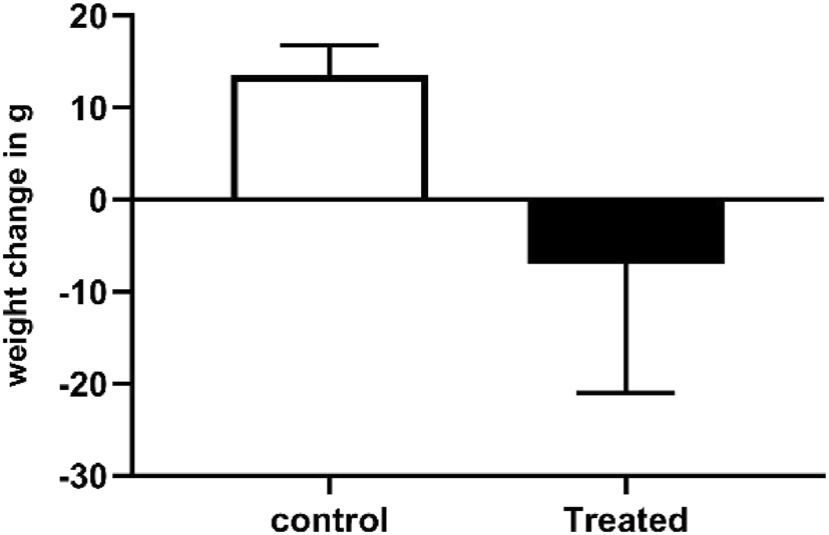
LD_50_ of the tested tetrazole derivative **4c** and body weight change of rats. *Results are expressed as mean ± S.E.M., n = 6, with *P* < 0.05 level of significance.

The tetrazole derivative **4c** was tested for its lethal toxicity against rats, and the mortality was measured. At first concentration, there was no mortality. At the second concentration, two rats died, and at the third concentration, 50% mortality was observed. Only the final concentration (2500 mg/kg) showed 50% mortality, as well as severe toxic signs including malaise and severe weight loss. That the median lethal dose for the tested compound is 2500 mg/kg (Fig. 14). To confirm the lethal toxicity of such concentration serum ALT, as well as liver bilirubin levels were investigated as vide infra.

### Biochemical assays

Serum ALT content was significantly higher in the treated group than the normal one by 95.6%. Indicating extreme liver toxicity, which was a result of 250 mg/kg compound. The total bilirubin is significantly (*P* < 0.0001) higher in the treated group that in the control by 94.8%. Also, the direct bilirubin is significantly (*P* < 0.05) higher in the treated group by only 49%. Eventually, the indirect bilirubin is significantly (*P* < 0.01) higher in the treated group by 272% (Table 6). The previous data indicate upon treatment the extreme liver toxicity and its inability to clear bilirubin properly. While the extreme increase in the indirect bilirubin may indicate other causes such as the inability of the liver to perform the glucuronidation process properly.

**Table 6:** Toxicity markers of the tested tetrazole derivative 4c*.

	Control	Treated
Change in weight	13.50 ± 0.96	− 7.083 ± 4.0
ALT enzyme activity	22.8 ± 31.24	44.6 ± 86.84
Total bilirubin	1.378 ± 0.10	2.67 ± 0.15
Direct bilirubin	1.12 ± 0.16	1.672 ± 0.16
Indirect bilirubin	0.25 ± 0.15	0.930 ± 0.14

*Results are expressed as mean ± S.E.M., n = 6, T test was applied With *P* < 0.05 level of significance.

### Computational chemical study

#### Density functional theory (DFT) study

The optimization of molecular structures for the produced compounds was carried out with DFT method using the hybrid *B3LYP* functional with 3-21G basis set. The molecular structures of compounds are superior to be formed to a stable geometry is known as optimization. Such energy of the structure is transported to a stationary point, the geometry of tetrazole derivatives was gradually optimized, and their energy were continuously decreased until the fluctuations in the molecule energy were minimized. The regions of highest electron density (HOMO) characterize the electrophilic-attacking sites, while the LUMO imitates the nucleophilic-attacked sites. The low values of the energy gap (Δ*E* = *E*_LUMO_–*E*_HOMO_) will render good inhibition efficiencies because the energy required to remove an electron from the last occupied orbital will be low^31,32^. DFT based on quantum chemical computation outline the structure optimization of the intermediate that reacted to afford the final product.

**Figure 15:**
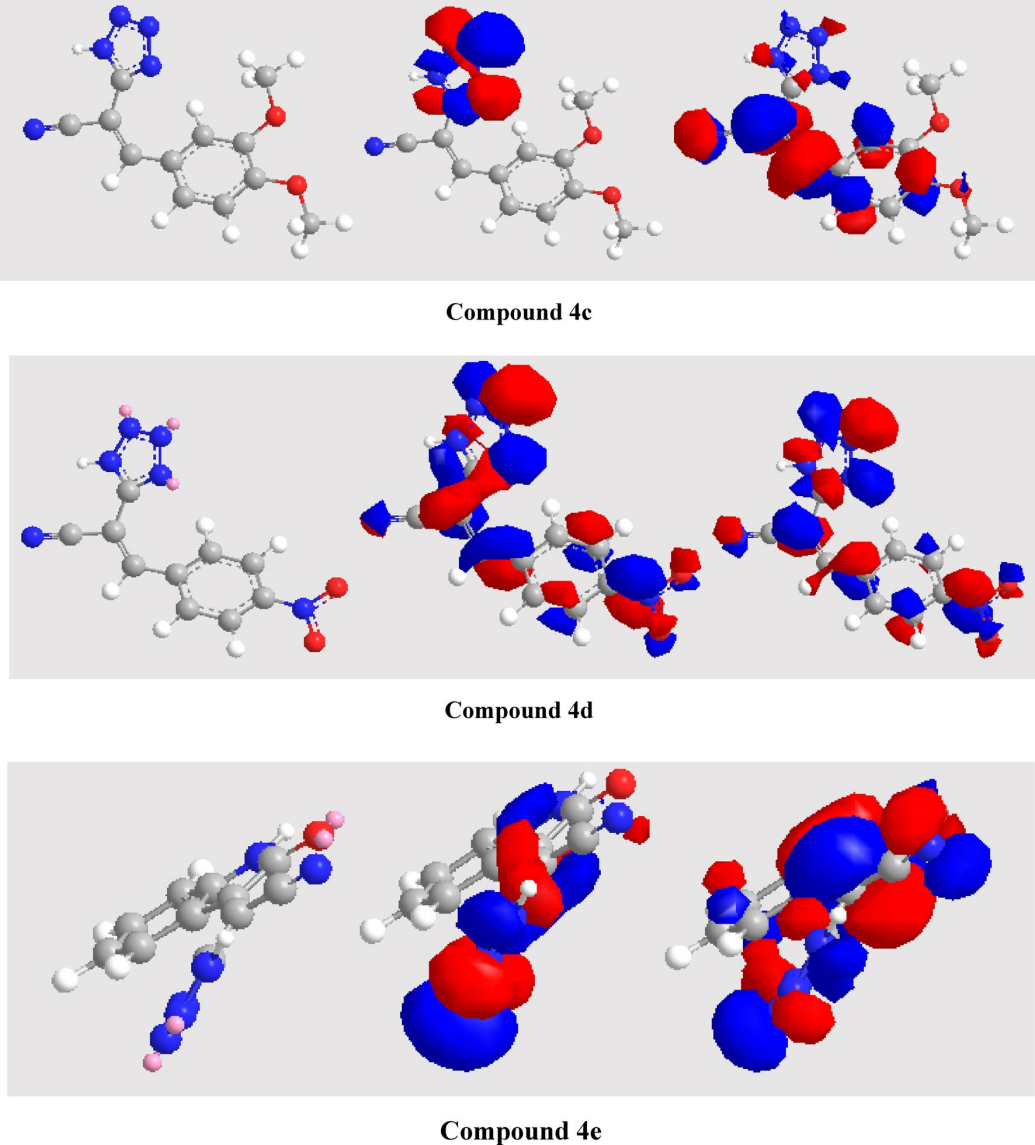
Optimized structures (left), HOMO (middle), and LUMO (right) for compounds **4c–e**. Atom color index: White H, Grey C, Blue N, and Red O.

So, DFT simulation helped to show the formation of the tetrazole derivative **4**. The structures have been supported by spectral analysis and analytical data. Quantum chemical parameters calculation with DFT method can be used for the calculations of the produced compounds (Table 7). The high E_HOMO_ are likely to indicate a strong tendency of the molecule to donate electrons. The dipole moments for tetrazole derivatives were agreed with an excellent explanation for the synthesized compounds. The optimized structures, HOMO’s and LUMO’s of the compounds **4c–e** were outlined in Fig. 15. In compounds **4c, 4d,** and **4e**, HOMOs are distributed over tetrazole unit, while LUMOs are focused on the benzene and nitrile moieties. The regions of highest electron density are generally the sites on which electrophiles attack the molecule and therefore the heteroatoms (O and N atoms) are generally the most active centers in these molecules.

**Table 7:** Global reactivity indices and energy level distribution of frontier orbitals.

Compd.	*E (eV)	*E*_HOMO_ (eV)	*E*_LUMO_ (eV)	Δ*E* (eV)	*µ* (Debye)	*Η* (eV)	*ς* (eV^−1^)	*μ*_*o*_ (eV)	*Ω* (eV)	*n* (eV^−1^)	*I*_*p*_ (eV)	*EA* (eV)	*X* (eV)
**4c**	36.297	− 6.278	− 4.094	2.184	2.935	1.092	0.916	− 5.186	12.31	0.081	6.278	4.094	5.18
**4d**	28.488	− 6.588	− 5.971	0.617	2.745	0.308	3.247	− 6.279	72.42	0.014	6.588	5.971	6.28
**4e**	35.918	− 6.390	− 5.521	0.869	0.505	0.434	2.304	− 5.955	40.85	0.024	6.390	5.521	5.95

*E: minimized energy (kcal/mol); *µ*: dipole moment; *η:* global hardness; *ς:* global softness; *μ*_*o*_*:* chemical potential; *ω:* global electrophilicity index; *n*: nucleophilicity index; *I*_*P*_: ionization potential; *EA*: electron affinity; *x*: electronegativity.

The calculated ΔE was compared with the theoretical reference data based on the corresponding experimental results in gas phase reaction. With ΔE being a criterion, three most typical and popular exchange–correlation functionals e.g., PW91 were systematically compared in terms of the typical synthesis in gas phase. In addition, the verification of geometrical and electronic properties of modeling the anticancer products, as well as the adsorption behaviors of typical probe reactants and solvents are also suggested for further screening proper functionalization. The present work provides general implications for how to choose a reliable exchange–correlation functional in the computational solvents and catalyst on reactant surface.

Quantum chemical parameters calculations utilizing DFT method used for the calculations of produced compounds are in good agreement with the anticancer efficiency (Table 7). The scavenging ability toward positive hole, tumor, radical, and oxygen removable not only depended upon *E*_HOMO_ values but also, the number of heteroatoms, electron distributions, surface area, and lipophilicity must be considered. The dipole moment, and softness (ϭ, eV^−1^), for most potent synthesized compounds carrying hydrophobic groups were agreed to an excellent correlation between oxidation inhibition efficiencies. Also, the compounds of higher binding energy are of higher potency due to the strong interaction between these compounds and the active sites of receptors.

#### Molecular dynamics simulation

**Figure 16:**
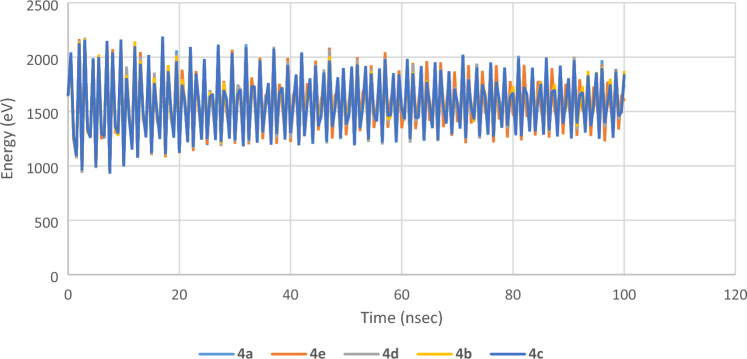
MD simulation of the synthesized compounds.

Molecular dynamics (MD) simulation is an effective method for studying the binding capability between ligands and protein. MD simulation is performed for the synthesized compounds (ligands) to investigate their tendency for binding with the active sites of protein^33^. Their binding tendency can be expressed in terms of the calculated binding energies (*E*_bind_) which are listed in Table 8. The values of *E*_bind_ calculated for these compounds reveal that their absolute magnitudes increase following the order: **4c** > **4b** > **4a** > **4e** > **4d**, and this is the same trend for the values of Δ*E* confirming that the best binding capability of the synthesized compounds with the active sites of protein, and hence the best potency, is observed for compound **4c**. This may be explained by such that the more polarizable the molecule, the better its interaction with protein. Figures 16, 17 (cf. Supporting information) demonstrates the binding energy of tetrazole derivatives **4a–e** with the active sites of protein. It is evident that the three compounds get bonded mainly by means of the tetrazole moiety, which is the moiety over which HOMOs are distributed. This implies that HOMO regions are the most active sites for interacting with protein.

**Table 8:** The binding energies (*E*_bind_) of the synthesized compounds **4a–e**.

Compounds	*E*_bind_ (eV)
**4a**	0.894
**4b**	1.082
**4c**	1.092
**4d**	0.3085
**4e**	0.4345

#### Pharmacokinetics studies

To direct the choice of selecting compounds from a huge collection of synthesized compounds in the early stages of drug discovery, biological activities, and development for an effective drug, ADME profiles including physicochemical properties, lipophilicity, and drug likeness of the synthesized compounds have been predicted^34–36^. The synthesized compounds **4c–e**, were found to meet Lipinski’s rule of five, having a total polar surface area (TPSA, Å) 96.71, 124.07, and 107.35, respectively, in addition to good lipophilicity, expressed by the consensus Log P_o/w_ which are in 1.20, 0.69, and 0.42, respectively. They exhibited a high GI absorption and were computed to possess a good bioavailability score (0.56), as displayed in Table 9. Their skin permeation (Log K_P_) parameter was − 6.94, − 6.93, and − 7.70, respectively, thus facilitating the accessibility of the bioactive molecules through the skin. The bioavailability of the synthesized compounds was also estimated based on their pink area on the radar chart (cf. Figs. 18, 19, 20 in Supporting information). These compounds were completely included in the pink area and justified their good predicted oral bioavailability.

**Table 9:** ADME properties of compounds **4c–e**.

Entry	Compounds		
	**4c**	**4d**	**4e**
Physicochemical properties/lipophilicity/druglikeness			
Molecular weight (g/mol)	257.25	242.19	238.20
Num. heavy atoms	19	18	18
Num. arom. heavy	11	11	11
Fraction Csp3	0.17	0.00	0.00
Num. rotatable bonds	4	3	1
Num. H-bond acceptor	6	6	5
Num. H-bond donors	1	1	2
Molar refractivity	67.09	62.93	64.43
TPSA (Å^2^)	96.71	124.07	107.35
Consensus log P_o/w_	1.20	0.69	0.42
Lipinskiˈs rule	Yes	Yes	Yes
Bioavailability score	0.56	0.56	0.56
Pharmacokinetics			
GI absorption	High	High	High
BBB permeant	No	No	No
P-gp substrate	No	No	No
CYP1A2 inhibitor	No	No	No
CYP2C19 inhibitor	No	No	No
CYP2C9 inhibitor	No	No	No
CYP2D6 inhibitor	No	No	No
CYP3A4 inhibitor	No	No	No
Log K_p_ (cm/s)	− 6.94	− 6.93	− 7.70

### Structure–activity relationship (SAR)

The inhibitory activity of the tested compounds could be linked to structure variation and modifications. The synthesized tetrazoles with sided electron-withdrawing head play a substantial role in the binding interactions with receptor subsites via van-der Waals interaction and hydrogen bonding, as well, facilitate *pi*-stacking interactions of the loop C aromatic residue with the side chain. The presence of aromatic moiety in these compounds increased hydrophobicity which improves their permeability into the cell membrane, therefore enhancing the antiproliferative activity. Besides, the analogs with extended conjugation had higher affinity to form a face-to-edge aromatic interaction with the receptor. Thus, it was revealed that the presence of two methoxy moieties (as in compound **4c**) increased the inhibitory effect against the cell lines which may be attributable to the formation of hydrogen bonding with receptor (cf. Fig. 6).

## Conclusion

A catalyst-free method was developed for the synthesis of structurally diverse substituted tetrazoles. A variety of 3-substituted 2-(1*H*-tetrazol-5-yl)acrylonitriles was obtained in good to excellent yields with great functional group compatibility. Our synthetic protocol included condensation of four benzaldehyde derivatives and/or indolin-2,3-dione with malononitrile and sodium azide to give five substituted tetrazoles. Molecular docking studies were carried out with the CSNK2A1 receptor to authenticate the epidermoid and colon cancer inhibition property of the synthesized molecules. The results concluded that compounds **4b, 4c,** and **4d** effectively inhibit the epidermoid cancer cell line among the other docked compounds. Also, the antimicrobial activity was evaluated against the bacteria: *K. pneumonia*, *S. aureus*, and the fungi: *Candida albicans*, as well as their antioxidant activity utilizing 2,2-diphenyl-1-picrylhydrazyl (DPPH) assay. In addition, the toxicity of the tetrazole derivative was assessed by determination of their approximate lethal dose fifty (LD_50_), calculated via an oral administration to rats. The antioxidant results indicated to tetrazoles exhibited great antioxidant properties even at very low doses. Molecular dynamics simulation has revealed that the synthesized derivatives tend to bind with protein following the same order shown by DFT study.

## Materials and methods

### General

Solvents and chemicals obtained from Sigma Aldrich, Merck, Fluka, and El-Nasr pharmaceutical chemicals companies were purified and dried by standard techniques. The reactions and the purity of the produced compounds were followed and checked by TLC using TLC aluminum sheets silica gel F_254_. Melting points were measured on a MEL-TEMP II electric melting point apparatus and are uncorrected. FTIR spectra (ν, cm^−1^) were run at Faculty of Science, Ain Shams University utilizing potassium bromide disks on FTIR Thermo Electron Nicolet iS10 infrared spectrometer (Thermo Fisher Scientific Inc., Waltham, MA, USA). ^1^H and ^13^C NMR spectra (δ, ppm) were run at 400 and 100 MHz on a BRUKER NMR spectrometer (BRUKER, Manufacturing & Engineering Inc., Anaheim, CA, USA) utilizing tetramethyl silane (TMS) as an internal standard in deuterated dimethyl sulfoxide. Mass spectra were recorded on a Shimadzu GC-MS-QP-1000 EX mass spectrometer (Shimadzu Scientific Instruments, Inc., USA) operating at 70 eV utilizing Thermo X-CALIBUR software, at Faculty of Science, Ain Shams University. Elemental analyses were recorded at Faculty of Science, Ain Shams University utilizing Perkin-Elmer 2400 CHN elemental analyzer. The microwave reactions were done by a Microsynth instrument type MA143 (Microwave flux). Sonication was performed in a Toshcon typical SW4 char (40 kHz and 150 W).

### General procedures for the synthesis of tetrazole derivatives **4a–e**

#### Method I: Using ethanol or glacial acetic acid as solvents

To a solution of aromatic aldehydes **1a–d** or indolin-2,3-dione **1e** (0.01 mol) and malononitrile **2** (0.01 mol) in ethanol or glacial acetic acid (20 mL), sodium azide **3** (0.015 mol dissolved in 1 mL of water) was added. The reaction mixture was refluxed as depicted in Table 1. After cooling, the products obtained were collected, dried, and recrystallized from the proper solvents.

#### Method II: Using EtONa, CAN, AlCl_3_, ZnCl_2_, and FeCl_3_ as a catalyst

To a mixture of aromatic aldehydes **1a–d** or indolin-2,3-dione **1e** (0.01 mol), malononitrile **2** (0.01 mol), and the catalyst; sodium ethoxide (EtONa) (0.015 mol dissolved in 5 mL of absolute ethanol), ceric ammonium nitrate (CAN), aluminum chloride (AlCl_3_), zinc chloride (ZnCl_2_), or ferric chloride (FeCl_3_) (0.015 mol) in ethanol (20 mL), sodium azide **3** (0.015 mol) was added. The reaction mixture was refluxed as depicted in Table 1. After cooling, the reaction mixture was poured onto ice/water. The separated products were collected and found to be the arylidine malononitrile derivatives **5a–e**.

#### Method III: Using sodium acetate as a catalyst

To a mixture of aromatic aldehydes **1a–d** or indolin-2,3-dione **1e** (0.01 mol), malononitrile **2** (0.01 mol), and catalyst; sodium acetate (0.015 mol) in ethanol (20 mL), sodium azide **3** (0.015 mol dissolved in 1 mL of water) was added, and the reaction mixture was refluxed as depicted in Table 1. The reaction mixture was then cooled and poured onto ice/water. The separated products were collected, washed with methanol, dried, and recrystallized from the proper solvents.

#### Method IV: Using ZnO, TiO_2_, NiFe_3_O_4_, CuFe_3_O_4_ (0.1 g) as a catalyst

To a mixture of aromatic aldehydes **1a–d** or indolin-2,3-dione **1e** (0.01 mol), malononitrile **2** (0.01 mol), and the catalyst; ZnO, TiO_2_, NiFe_3_O_4_, CoFe_2_O_4_, or CuFe_3_O_4_ (0.015 mol) in ethanol (20 mL), sodium azide **3** (0.015 mol dissolved in 1 mL of water) was added, and the reaction mixture was refluxed as depicted in Table 1. The reaction mixture was cooled and then poured onto ice/water. The products separated were collected, washed with methanol, dried, and then recrystallized from the proper solvents.

#### Method V: Using ultrasonic irradiation

A mixture of aromatic aldehydes **1a–d** or indolin-2,3-dione **1e** (0.01 mol), malononitrile **2** (0.01 mol), and sodium azide **3** (0.015 mol) in dimethyl formamide (2 mL) was sonicated as depicted in Table 1. The reaction mixture was concentrated under reduced pressure. The crude substance was triturated with methanol, filtered off, and found to be the arylidine malononitrile derivatives **5a–e**.

#### Method VI: Using microwave irradiation

A mixture of aromatic aldehydes **1a–d** or indolin-2,3-dione **1e** (0.01 mol), malononitrile **2** (0.01 mol), and sodium azide **3** (0.015 mol) in dimethyl formamide (2 mL) was added to the reaction vessel of the monomodal Emrys™ Creator microwave synthesizer and allowed to react under microwave irradiation at 200–400 W power (Table 1). The automatic mode stirring helped with mixing and uniformly heating the reactants. The reaction vessel was cooled to room temperature. The products separated were collected, washed with methanol, dried, and then recrystallized from the proper solvents.

#### Method VII: Neat condition

In a round bottomed flask, aromatic aldehydes **1a–d** or indolin-2,3-dione **1e** (0.01 mol), malononitrile **2** (0.01 mol), and sodium azide **3** (0.015 mol) were fused in a sand bath at 140–200 °C (Table 1). After cooling, the products were triturated with methanol, filtered off, and then recrystallized from the proper solvents to give the tetrazole derivatives **4a–e**.

##### 3-Phenyl-2-(1H-tetrazol-5-yl)acrylonitrile (**4a**)

Paly-yellow crystals, mp. 170–172 °C^5^. IR (KBr, ν, cm^−1^): 3590 (NH), 2209 (C≡N). ^1^H NMR (400 *MHz*, DMSO-*d*_6_, δ, ppm): 3.68 (*br*.s, 1H, NH, exchangeable), 7.24–7.60 (m, 3H, Ar–H), 7.95 (d, 2H, Ar–H, *J* = 7.45 *Hz*), 8.45 (s, 1H, CH=). Anal. Calcd. for C_10_H_7_N_5_ (197.20): C, 60.91; H, 3.58; N, 35.51; Found: C, 60.80; H, 3.52; N, 35.53%.

##### 3-(4-Methoxyphenyl)-2-(1H-tetrazol-5-yl)acrylonitrile (**4b**)

Yellow crystals, mp. 153–155 °C^5^. IR (KBr, ν, cm^−1^): 3320 (NH), 2235 (C≡N), 1605 (C=N). ^1^H NMR (400 *MHz*, DMSO-*d*_6_, δ, ppm): 3.45 (*br*.s, 1H, NH, exchangeable), 7.83 (d, 2H, Ar–H, *J* = 7.5 Hz), 7.90 (d, 2H, Ar–H, *J* = 7.5 Hz), 8.01 (s, 1H, CH=). Anal. Calcd. for C_11_H_9_N_5_O (227.23): C, 58.14; H, 3.99; N, 30.82; Found: C, 58.07; H, 3.90; N, 30.80%.

##### 3-(3,4-Dimethoxyphenyl)-2-(1H-tetrazol-5-yl)acrylonitrile (**4c**)

Yellow crystals, mp. 190–192 °C^5^. IR (KBr, ν, cm^−1^): 3315 (NH), 2230 (C≡N), 1603 (C=N). ^1^H NMR (400 *MHz*, DMSO-*d*_6_, δ, ppm): 3.46 (*br*.s, 1H, NH, exchangeable), 3.77 (s, 3H, OCH_3_), 3.82 (s, 3H, OCH_3_), 7.61–7.72 (m, 2H, Ar–H), 7.87 (s, 1H, Ar–H), 8.20 (s, 1H, CH=). EIMS (70 eV, *m/z*, %): 260.57 (M^+⋅^+2, 16.63), 255.26 (M^+⋅^−2, 42.19), 254.16 (48.63), 238.17 (100), 226.27 (67.78), 211.16 (55.50), 180.27 (54.37), 177.16 (53.80), 148.77 (53.02), 127.99 (61.40), 120.66 (86.42), 105.06 (33.28), 69.50 (87.96). Anal. Calcd. for C_12_H_11_N_5_O_2_ (257.25): C, 56.03; H, 4.31; N, 27.22; Found: C, 55.97; H, 4.26; N, 27.25%.

##### 3-(4-Nitrophenyl)-2-(1H-tetrazol-5-yl)acrylonitrile (**4d**)

Beige crystals, mp. 167–169 °C^5^. IR (KBr, ν, cm^−1^): 3354 (NH), 2253 (C≡N), 1608 (C=N), 1573, 1436 (NO_2_). ^1^H NMR (400 *MHz*, DMSO-*d*_6_, δ, ppm ): 3.52 (*br*.s, 1H, NH, exchangeable), 8.18 (d, 2H, Ar–H, *J* = 7.5 Hz), 8.36 (d, 2H, Ar–H, *J* = 7.5 Hz), 8.48 (s, 1H, CH=). Anal. Calcd. for C_10_H_6_N_6_O_2_ (242.20): C, 49.59; H, 2.50; N, 34.70; Found: C, 49.51; H, 2.44; N, 34.67%.

##### 2-(2-Oxoindolin-3-ylidene)-2-(1H-tetrazol-5-yl)acetonitrile (**4e**)

Red crystals, mp. 220–222 °C. IR (KBr, ν, cm^−1^): 3375, 3240 (NH), 2219 (C≡N), 1714 (C=O). ^1^H NMR (400 *MHz*, DMSO-*d*_6_, δ, ppm): 7.36–6.84 (m, 4H, Ar–H), 9.09 (*br*.s, 1H, NH, exchangeable), 10.84 (*br*.s, 1H, NH indoline, exchangeable). ^13^C NMR (100 *MHz*, DMSO-*d*_6_, δ, ppm): 166.93 156.77, 143.50, 134.94, 133.27, 128.67, 121.80, 121.21, 116.71, 110.18, 107.36. EIMS (70 eV, *m/z,* %): 240.96 (M^+⋅^+2, 26.72), 236.82 (M^+⋅^−2, 11.15), 210.27 (12.56), 202.37 (15.20), 178.96 (26.93), 166.27 (27.01), 156.03 (53.49), 154.24 (48.82), 137.99 (46.83), 121.50 (40.19), 107.23 (20.14), 82.85 (35.52), 75.06 (42.31), 70.27 (41.32), 41.65 (100). Anal. Calcd. for C_11_H_6_N_6_O (238.21): C, 55.46; H, 2.54; N, 35.28; Found: C, 55.37; H, 2.50; N, 35.30%.

### Preparation of catalyst

#### Preparation of spinel ferrites (CuFe_2_O_4_, CoFe_2_O_4_, NiFe_2_O_4_)

The preparation of copper ferrite active phase and magnetic recycling responsible part of the composite which was selected to be a Ferro spinel which are known for their stability, super paramagnetism, availability and high catalytic activity regarding nitro phenols reduction. First, a three Ferro spinel samples (namely, copper ferrite, cobalt ferrite, and nickel ferrite) were selected from a pool of many ferro-spinels from which the copper ferrite was selected after some comparative trials for being the most active toward the target reaction. Where a simple co-precipitation method was used to prepare all three spinels for this simple comparison in the activity toward the desired reaction, resulting in copper ferrite being the most active of the three selected spinels due to the presence of copper ions in its formula and crystal lattice leading to an improved catalytic activity toward nitro phenols reduction. The preparation process was further improved by the addition of a polymer to further reduce the copper ferrite size and catalytic activity. The polymer was also selected from a pool of four polymers for comparison and optimization for the selected model reaction, being polyethylene glycol (PEG), where the most effective polymer in stabilizing the formed particulates was PEG which did not only improve stability and size but also introduced further surface defects improving the catalytic activity and was optimized by using different amounts from the polymer to stabilize the formed particles. Where the 2% weight ratio was selected for the comparison between different polymers.

The simple preparation method done using a mixture containing (1:2) ratio of (copper, cobalt, or nickel) and ferric ions which was stirred together first, and the appropriate amount of the polymer was then added to the mixture and stirred for 1 h. Sodium hydroxide solution (1 M) was then added dropwise to begin the precipitation of the copper-iron double hydroxide until the mixture pH reached a basic pH ~ 10. The mixture was then filtered off and washed with copious amounts of distilled water to ensure the removal of chloride ions (using silver nitrate) and the excess sodium hydroxide. The obtained slurry was then dried in and oven at 100 °C overnight to obtain the powder, which was then crushed into a mortar to ensure size uniformity. The obtained fine powder was then calcinated at 600 °C for 3 h to ensure polymer removal and the complete transformation of the formed copper-iron double hydroxide to a spinel. The calcinated powder was then crushed again to obtain the final ferrite spinel in powder form.

#### Preparation of titanium dioxide (TiO_2_) nanoparticles

Titanium tetraisopropoxide (20 mL) was dissolved in isopropanol (80 mL), then hydrolyzed by the slow addition of distilled water. The mixture obtained was kept for stirring for 2 h and then left for two days for complete hydrolysis. The precipitate formed was filtered off, washed, and then dried at 100 °C overnight and calcined at 600 °C for 2 h^37^. X-ray diffraction (XRD) analysis was performed to assess the nature and size of titania crystalline phases. The diffraction pattern of titania can be illustrated in Fig. 21 (cf. Supporting information) to explore the influence of the bio-template on titania crystalline features. On examining that Fig. 21, one can observe several titania crystalline and 75 [JCPDS No. 71-1167 were a = 3.786 Å and c = 9.507 Å] revealing the existence of anatase phase and other peaks at 2θ = 27.4°, 39°, 41°, and 44° [JCPDS No. 88-1175, a = 0.4.5 Å and c = 2.940 Å] referred to rutile phase. The trans-mission electron microscopy (TEM) is considered a powerful tool in determining the nanostructure of the prepared sample. It is clear that titania nanoparticles exist in a spherical structure with size about 25 nm, (cf. Fig. 22 in Supporting information).

#### Preparation of zinc oxide (ZnO) nanoparticles

To a solution of zinc chloride (30 mmol in 100 mL water), cyclohexane (50 mL) and ethanol (50 mL) were added. The mixture was stirred for 15 min. Then, an aqueous solution of ammonia (40%) was added to the solution dropwise with stirring for 10 min. The precipitate formed was washed with water several times and then dried in an oven at 100 °C for 1 h, followed by calcinations at 500 °C for 2 h^38^. The ZnO-NPs were assessed by XRD for their crystalline nature. The diffraction pattern showed four main peaks at 2*θ* values of 31.8, 34.5, 36.27, 47.5, 56.7, 62.8, and 67.5 (cf. Fig. 23). The mean particle size of zinc nanoparticles was calculated using Scherrer’s equation: (D = K*λ*/*β* cos *θ*), which are mostly relevant for the ZnO structure. Observable line broadening in the XRD peaks indicates that the material which is synthesized is in the nm range. TEM analysis was performed to better recognize crystalline properties and size of the synthesized nanoparticles. The TEM images (cf. Fig. 24) of ZnO confirmed that the particles are hexagonal with little thickness variation. This figure shows that the majority of the ZnO-NPs are hexagonal in form, with typical particle sizes of 100 nm.

### Biology

#### Molecular docking

As known, CSNK2A1 enzyme was inhibited by the ligand ERB-041, [2-(3-fluoro-4-hydroxyphenyl)-7-vinylbenzo[*d*]oxazol-5-ol], the molecular docking study has been executed on the synthesized tetrazole derivatives as unprecedented ligand in inhibition of CSNK2A1 which was restored from protein data bank (PDB: 6QY7) (http://www.rcsb.org/pdb) as a reference ligand for docking score. Al enzymes were fitted for docking study by reconnecting the bond broken, fixing the potential, and adding the lost hydrogen. Moreover, the known ligand ERB-041 was exchanged by our synthesized tetrazole derivatives at the identified active sites.

#### Cytotoxic activity

The cancer cell lines: A431 (Epidermoid carcinoma), HCT116 (colon cancer), and the normal cell line (BJ-1) were obtained from Karolinska Institutes, Sweden. As a gift from Dr. Stig Linder. Each one of them was maintained in a suitable medium with 10% FBS. Cell lines were incubated at 37 °C in 5% CO_2_ and 95% humidity. Cells were sub-cultured using trypsin 0.15%^29^.

#### Cell viability assay

For A431 and HCT116 cell lines, cell seeding was conducted at count 10 * 10^3^ cells/well (96 well plate), while for BJ-1, cell seeding was conducted at count 50 * 10^3^. After 24 h, the medium was discarded, and a new serum free medium was added with the tested compound at different concentrations of 12.5, 25, 50, and 100 µg/mL. After 24 h of incubation, cell viability was determined using MTT (3-(4-,5-dimethylthiazol-2-yl)-2,5-diphenyltetrazolium bromide) assay^29^. Doxorubicin was used as a positive control and DMSO was used as a negative one. The exact IC_50_ was calculated with calculated using SPSS.

#### Determining the minimal inhibitory concentrations (MIC)

The minimal inhibitory concentrations (MIC) of the tested compound were determined by a modified version of Clinical and Laboratory Standards Institute (CLSI) protocol. The procedure involves preparing twofold dilutions of the tested compounds (from 100 to 0.05 mg/mL) in a liquid growth medium dispensed in a 96-well microplate. The tested organisms were *Klebsiella pneumonia* ATCC 700603, *Staphylococcus aureus* ATCC 29213, and *Candida albicans* (as a representative for Gram negative bacteria, Gram positive bacteria and fungi, respectively). Each well is inoculated with a microbial inoculum prepared in the same medium and adjusted to 0.5 McFarland scale (1–5 × 10^5^ CFU/mL). After well-mixing, the inoculated microplates are incubated for 24 h at 37 °C and the lowest concentration of the tested compound that prevented microbial growth (showed no turbidity) was measured visually and recorded as its MIC. The study was approved by the Research Ethics Committee at Ain Shams University and was performed in accordance with the guidelines of the National Institute of Health (NIH).

#### Radical scavenging activity prior to animal testing

Prior to any animal testing, the compound antioxidant capacity was evaluated against ascorbic acid using 2,2-diphenyl-1-picrylhydrazyl (DPPH) assay^30^. DPPH⋅ has a characteristic absorbance at 515 nm which disappears after its reduction by an antiradical compound. The reduction in DPPH thus can be monitored by measuring the decrease in its absorbance at 515 nm during the reaction.

#### Calculation of LD_50_ in male albino rats

- *Animals*: A total of 24 male albino rats (*Rattus norvegicus*) were randomly selected for the present study, each weighing 160 ± 20 g. These rats were housed in animal houses with a natural 12 h. Dark/light cycle at 27 °C. They had free access to food and tap water. All animals were healthy and were allowed to adapt to the laboratory conditions for at least a week prior to the start of the experiment. The rats were allotted into two groups, (1) the control group (n = 6), received only tap water through the gavage. (2) Treated group (n = 18), which received the tetrazole derivative dose for LD_50_ calculation. The LD_50_ was determined following the OECD 423 guidelines.
  The tetrazole derivative was administered orally by gavage. First, at concentration of 500 mg/kg, then 2000 mg/kg and eventually at 2500 mg/kg (for each concentration, a total of 6 rats received the dose).
  Animals were observed individually after dosing at least once every 30 min. For the following 5 h after intubation, and twice a day along the following 14 days. Afterwards, the following parameters were measured.
- *Body weight change calculation:* The body weights of rats were recorded at the beginning and the end of the experiment, then the change in body weight was calculated by subtracting the rats body weights at the beginning of the experiment from that at the end of it. The body weights of rats were recorded at the beginning and the end of the experiment, then the change in body weight was calculated by subtracting the rats body weights at the beginning of the experiment from that at the end of it.
  $$\mathrm{Body\; weight \;change}=\mathrm{body \; weight \; at \; the\; beginning \; of\; the\; experiment}-\mathrm{body \;weight \; at\; the\; end \;of\; it}.$$
- *Biochemical methods in serum:* The LD_50_ calculation was confirmed through measuring ALT activity and bilirubin content in serum.
  Testing for alanine-amino transferase (ALT): ALT was measured through the kinetic method according to the International Federation of Clinical Chemistry (1986). The assay principle depends on assaying the rate of NADH oxidation, which is proportional to the reduction in Absorbance at 340 nm over time.
  Bilirubin toxicity: was measured through the colorimetric method provided by the BioMed-Diagnostics bilirubin kit. The principle depends on the fact that total bilirubin reacts in acidic medium with diazotized sulfanilic acid to form a red colored azobilirubin and the intensity of the color produced is directly proportional to the amount of bilirubin present.

### Ethics declaration

This study was approved by the Research Ethics Committee at Ain Shams University (Approval code: ASU-SCI/CHEM/2023/5/6) and was performed in accordance with the guidelines of the National Institute of Health (NIH). All methods are reported in accordance with ARRIVE guidelines.

### Supplementary Information

Supplementary Information 1.

Supplementary Information 2.

Supplementary Information 3.

## Data Availability

All data generated or analyzed during this study are included in this published article and its supplementary information files.
