# Supplementary material for: One-pot synthesis, computational chemical study, molecular docking, biological study, and in silico prediction ADME/pharmacokinetics properties of 5-substituted 1H-tetrazole derivatives
Source: Sci Rep. 2023 Oct 19;13:17869. doi: 10.1038/s41598-023-44615-4 (PMC10587066; doi:10.1038/s41598-023-44615-4)
Supplement: Supplementary file 2 — Supplementary Information 2. [file 41598_2023_44615_MOESM2_ESM.pdf]

One-Pot Synthesis, Computational Chemical Study, Molecular Docking, Biological Study, and *in Silico* Prediction ADME/Pharmacokinetics Properties of 5-Substituted 1*H*-Tetrazole Derivatives

Ahmed El-Sewedy[1], Eman A. El-Bordany[1], Naglaa F. H. Mahmoud[1], Kholoud A. Ali[2], Sayed K. Ramadan[1,*]

[1] Chemistry Department, Faculty of Science, Ain Shams University, Cairo 11566, Egypt

[2] Zoology Department, Faculty of Science, Ain Shams University, Cairo 11566, Egypt

**E*-mail: sayed.karam2008@sci.asu.edu.eg

Supporting information:

Cont. Table 3. Cytotoxic activity of compound **4c** against the tested cell lines.

		***In-vitro* cytotoxicity**											
Cell lines		*BJ-1*				*A431*				*HCT116*			
Concn.		100	50	25	12.5	100	50	25	12.5	100	50	25	12.5
Antitumor effect	1st test	42.9	39.02	29	0	70.5	63.2	60.7	2.53	21	11.2	0	0
	2nd test	50.2	41.03	41.03	6.41	65.8	71.4	66.4	4.11	22	9.6	0	0
	3rd test	52.9	38.02	42.6	14.1	89.1	54.06	37.3	25.3	19.7	10.4	0	0
	Average	48.667	39.357	37.543	6.837	75.133	62.887	54.8	10.647	20.9	10.4	0	0
	**IC_{50} (μg/mL)**	**92.045**				**44.77**				**201.45**			

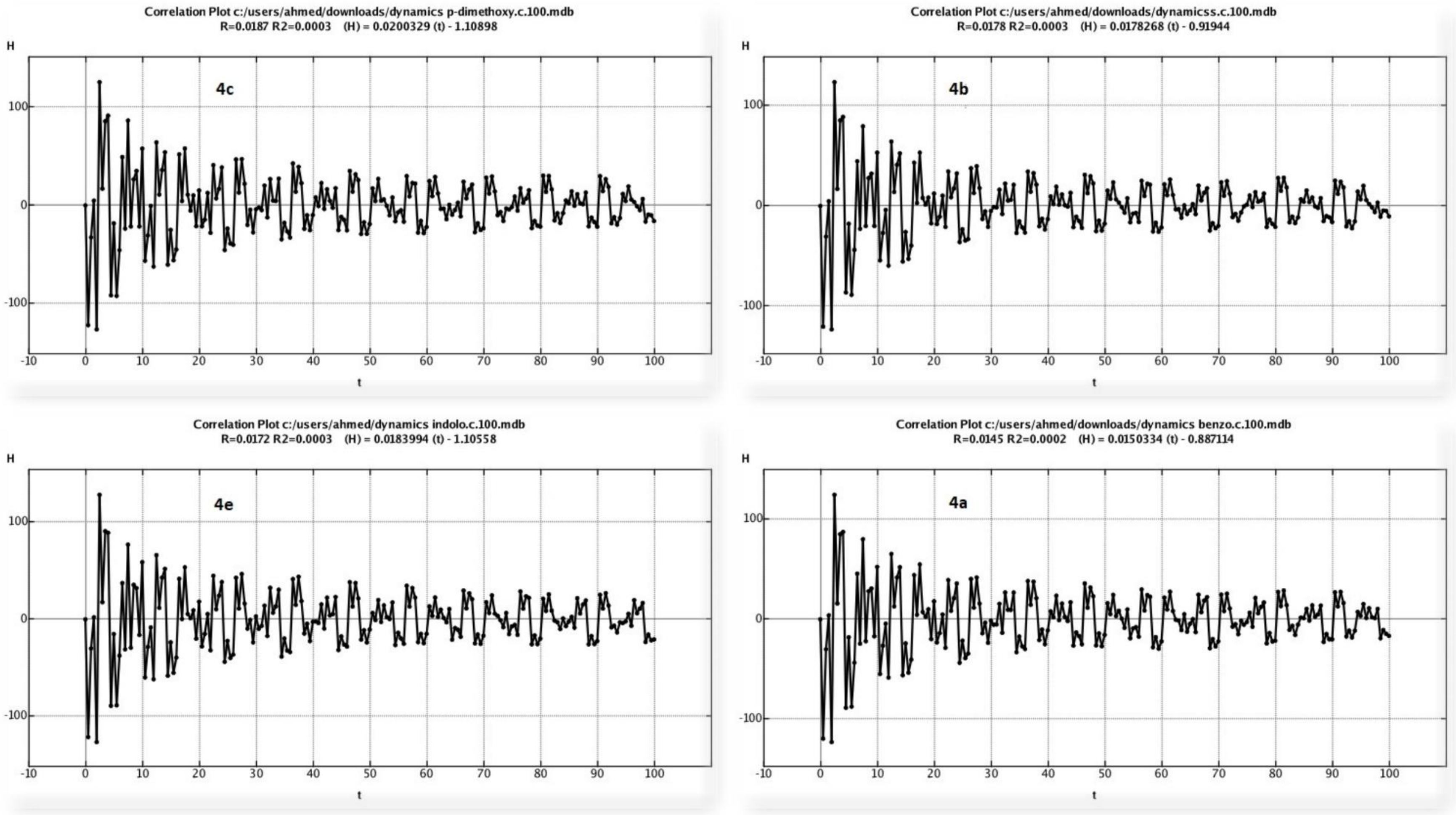

Fig. 17. MD simulation of the synthesized compounds **4a-e**.

Molecule 1

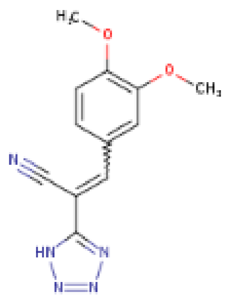

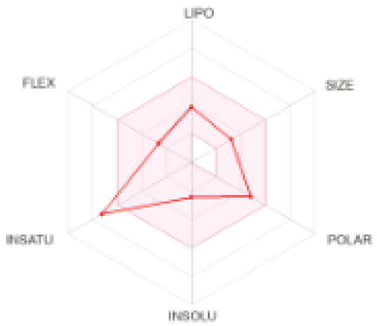

SMILES N#C/C(=C\c1ccc(c(c1)OC)OC)/c1nnn[nH]1

Physicochemical Properties	
Formula	C12H11N5O2
Molecular weight	257.25 g/mol
Num. heavy atoms	19
Num. arom. heavy atoms	11
Fraction Csp3	0.17
Num. rotatable bonds	4
Num. H-bond acceptors	6
Num. H-bond donors	1
Molar Refractivity	67.09
TPSA	96.71 Å²

Lipophilicity	
Log $P_{o/w}$ (iLOGP)	1.25
Log $P_{o/w}$ (XLOGP3)	1.31
Log $P_{o/w}$ (WLOGP)	1.17
Log $P_{o/w}$ (MLOGP)	0.57
Log $P_{o/w}$ (SILICOS-IT)	1.71
Consensus Log $P_{o/w}$	1.20

Water Solubility	
Log *S* (ESOL)	-2.42
Solubility	9.68e-01 mg/ml ; 3.76e-03 mol/l
Class	Soluble
Log *S* (Ali)	-2.94
Solubility	2.94e-01 mg/ml ; 1.14e-03 mol/l
Class	Soluble
Log *S* (SILICOS-IT)	-3.44
Solubility	9.28e-02 mg/ml ; 3.61e-04 mol/l
Class	Soluble

Pharmacokinetics	
GI absorption	High
BBB permeant	No
P-gp substrate	No
CYP1A2 inhibitor	No
CYP2C19 inhibitor	No
CYP2C9 inhibitor	No
CYP2D6 inhibitor	No
CYP3A4 inhibitor	No
Log K_p (skin permeation)	-6.94 cm/s

Druglikeness	
Lipinski	Yes; 0 violation
Ghose	Yes
Veber	Yes
Egan	Yes
Muegge	Yes
Bioavailability Score	0.56

Medicinal Chemistry	
PAINS	0 alert
Brenk	1 alert: conjugated_nitrile_group
Leadlikeness	Yes
Synthetic accessibility	2.87

Fig. 18. ADME prediction of compound **4c**.

Molecule 2

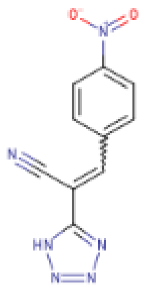

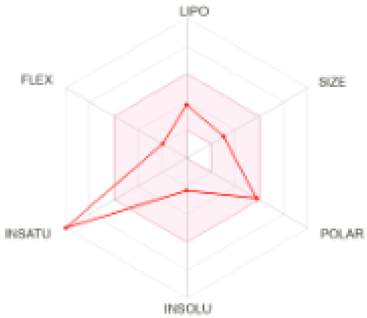

SMILES N#C/C(=C\c1ccc(cc1)[N+](=O)[O-])/c1nnn[nH]1

Physicochemical Properties	
Formula	C10H6N6O2
Molecular weight	242.19 g/mol
Num. heavy atoms	18
Num. arom. heavy atoms	11
Fraction Csp3	0.00
Num. rotatable bonds	3
Num. H-bond acceptors	6
Num. H-bond donors	1
Molar Refractivity	62.93
TPSA	124.07 Å²

Lipophilicity	
Log $P_{o/w}$ (iLOGP)	0.71
Log $P_{o/w}$ (XLOGP3)	1.19
Log $P_{o/w}$ (WLOGP)	1.06
Log $P_{o/w}$ (MLOGP)	0.96
Log $P_{o/w}$ (SILICOS-IT)	-0.49
Consensus Log $P_{o/w}$	0.69

Water Solubility	
Log *S* (ESOL)	-2.35
Solubility	1.09e+00 mg/ml ; 4.51e-03 mol/l
Class	Soluble
Log *S* (Ali)	-3.39
Solubility	9.83e-02 mg/ml ; 4.06e-04 mol/l
Class	Soluble
Log *S* (SILICOS-IT)	-2.57
Solubility	6.50e-01 mg/ml ; 2.69e-03 mol/l
Class	Soluble

Pharmacokinetics	
GI absorption	High
BBB permeant	No
P-gp substrate	No
CYP1A2 inhibitor	No
CYP2C19 inhibitor	No
CYP2C9 inhibitor	No
CYP2D6 inhibitor	No
CYP3A4 inhibitor	No
Log K_p (skin permeation)	-6.93 cm/s

Druglikeness	
Lipinski	Yes; 0 violation
Ghose	Yes
Veber	Yes
Egan	Yes
Muegge	Yes
Bioavailability Score	0.56

Medicinal Chemistry	
PAINS	0 alert
Brenk	3 alerts: conjugated_nitrile_group, nitro_group, oxygen-nitrogen_single_bond
Leadlikeness	No; 1 violation: MW<250
Synthetic accessibility	2.71

Fig. 19. ADME prediction of compound **4d**.

Molecule 3

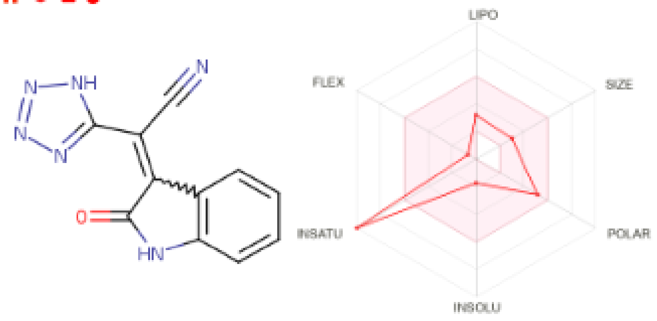

SMILES N#C/C(=C/1\C(=O)Nc2c1cccc2)/c1nnn[nH]1

Physicochemical Properties	
Formula	C11H6N6O
Molecular weight	238.20 g/mol
Num. heavy atoms	18
Num. arom. heavy atoms	11
Fraction Csp3	0.00
Num. rotatable bonds	1
Num. H-bond acceptors	5
Num. H-bond donors	2
Molar Refractivity	64.43
TPSA	107.35 Å²

Lipophilicity	
Log $P_{o/w}$ (iLOGP)	0.58
Log $P_{o/w}$ (XLOGP3)	0.07
Log $P_{o/w}$ (WLOGP)	0.01
Log $P_{o/w}$ (MLOGP)	0.35
Log $P_{o/w}$ (SILICOS-IT)	1.07
Consensus Log $P_{o/w}$	0.42

Water Solubility	
Log *S* (ESOL)	-1.75
Solubility	4.26e+00 mg/ml ; 1.79e-02 mol/l
Class	Very soluble
Log *S* (Ali)	-1.88
Solubility	3.15e+00 mg/ml ; 1.32e-02 mol/l
Class	Very soluble
Log *S* (SILICOS-IT)	-3.62
Solubility	5.69e-02 mg/ml ; 2.39e-04 mol/l
Class	Soluble

Pharmacokinetics	
GI absorption	High
BBB permeant	No
P-gp substrate	No
CYP1A2 inhibitor	No
CYP2C19 inhibitor	No
CYP2C9 inhibitor	No
CYP2D6 inhibitor	No
CYP3A4 inhibitor	No
Log K_p (skin permeation)	-7.70 cm/s

Druglikeness	
Lipinski	Yes; 0 violation
Ghose	Yes
Veber	Yes
Egan	Yes
Muegge	Yes
Bioavailability Score	0.56

Medicinal Chemistry	
PAINS	0 alert
Brenk	2 alerts: conjugated_nitrile_group, michael_acceptor_1
Leadlikeness	No; 1 violation: MW<250
Synthetic accessibility	2.60

Fig. 20. ADME prediction of compound **4e**.

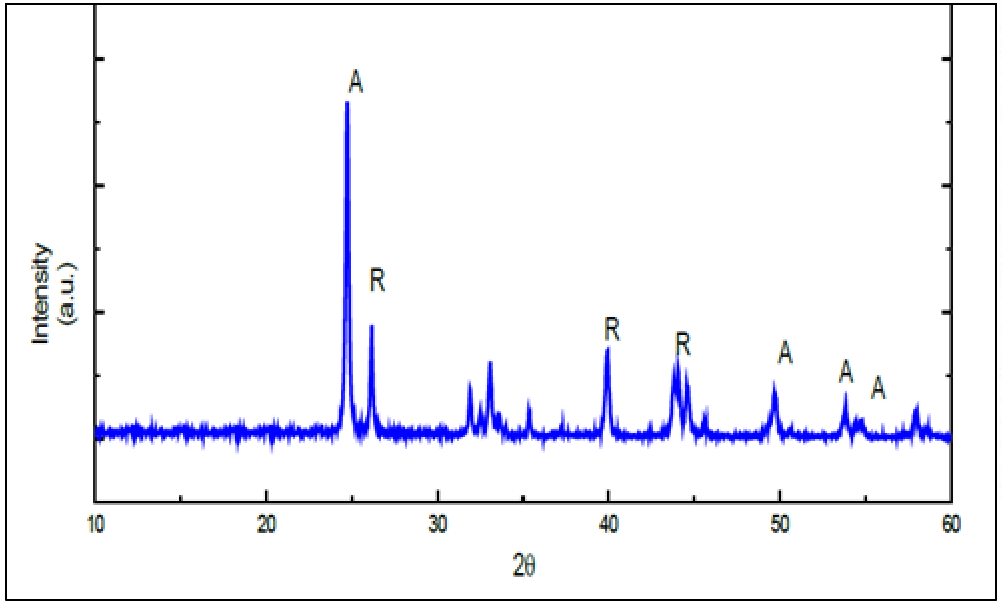

Fig. 21. XRD pattern of TiO_2 nanoparticles.

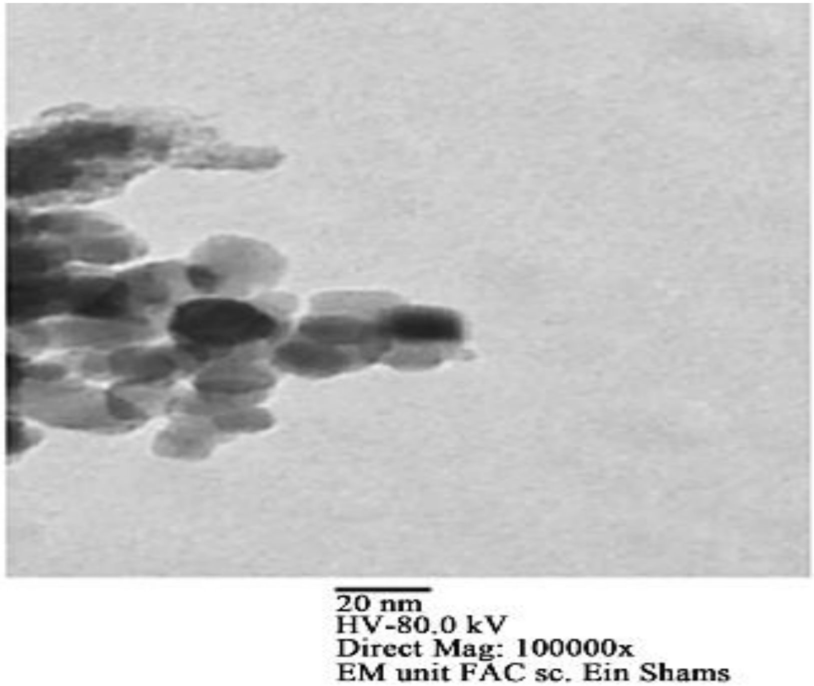

Fig. 22. TEM micrographs of TiO_2 nanoparticles.

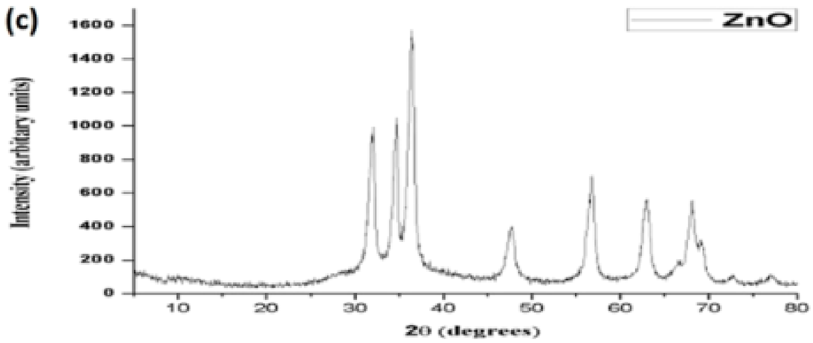

Fig. 23. XRD pattern of ZnO nanoparticles.

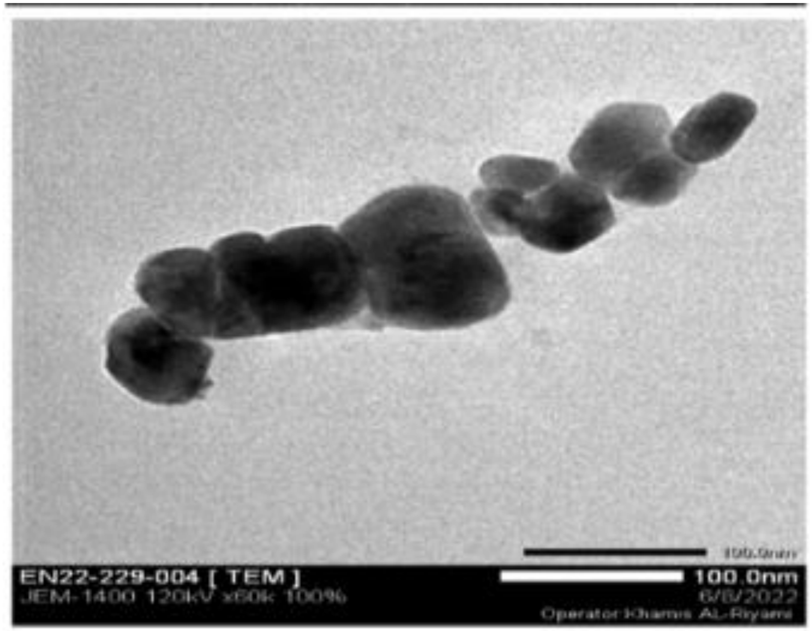

Fig. 24. TEM micrographs of ZnO nanoparticles.

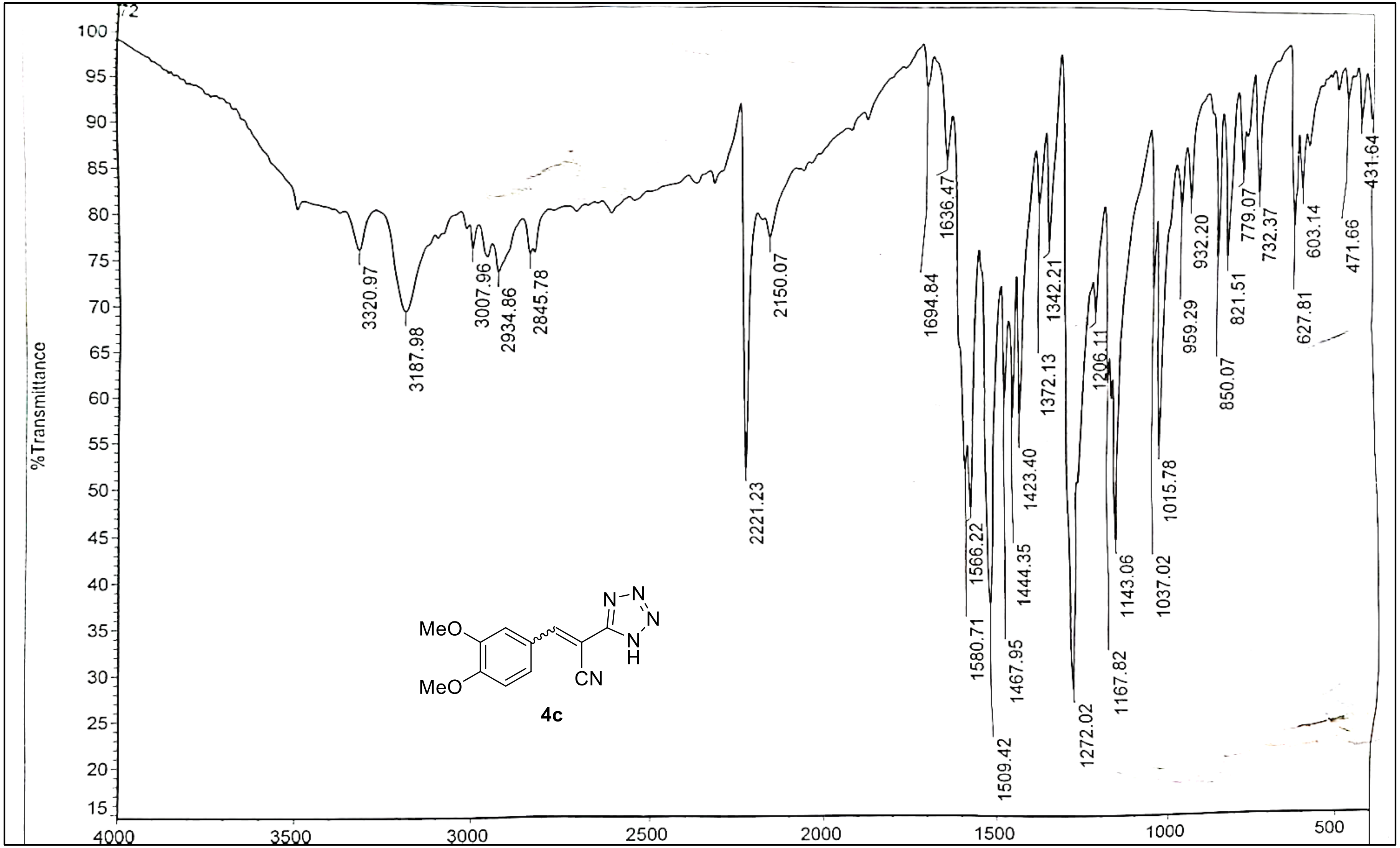

IR spectrum of compound **4c**.

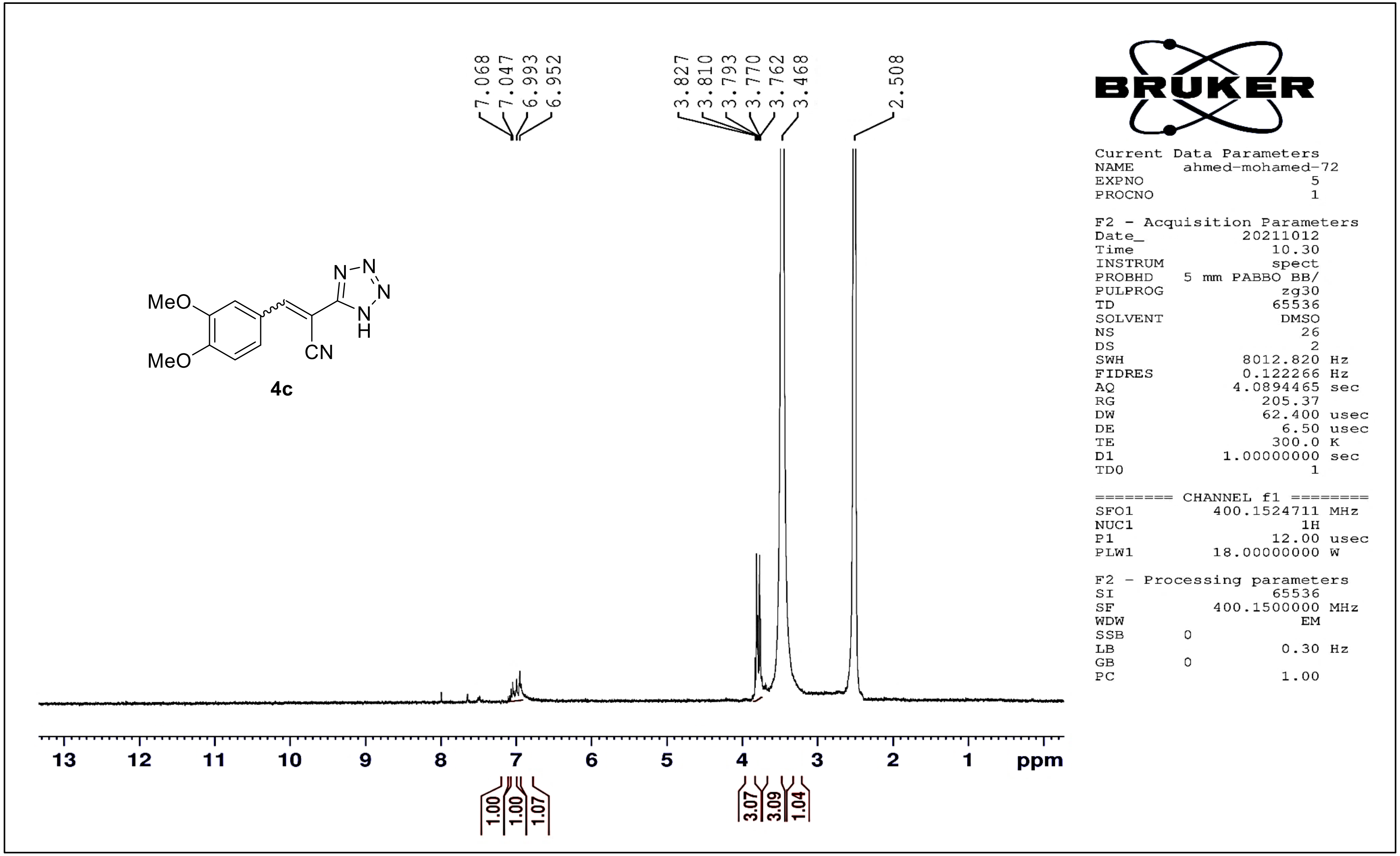

^{1}H NMR spectrum (DMSO-d_6) of compound **4c**.

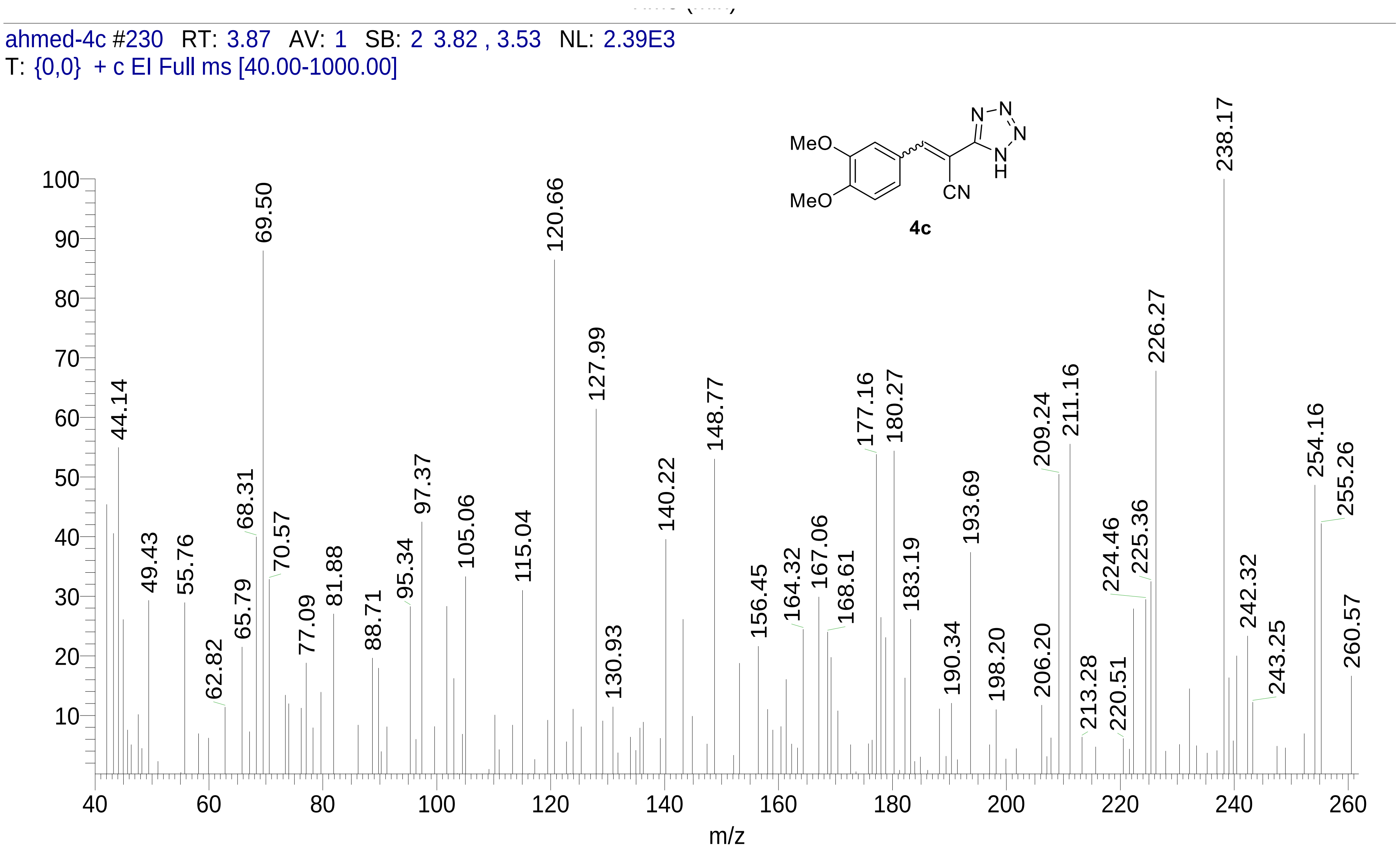

Mass spectrum of compound **4c**.

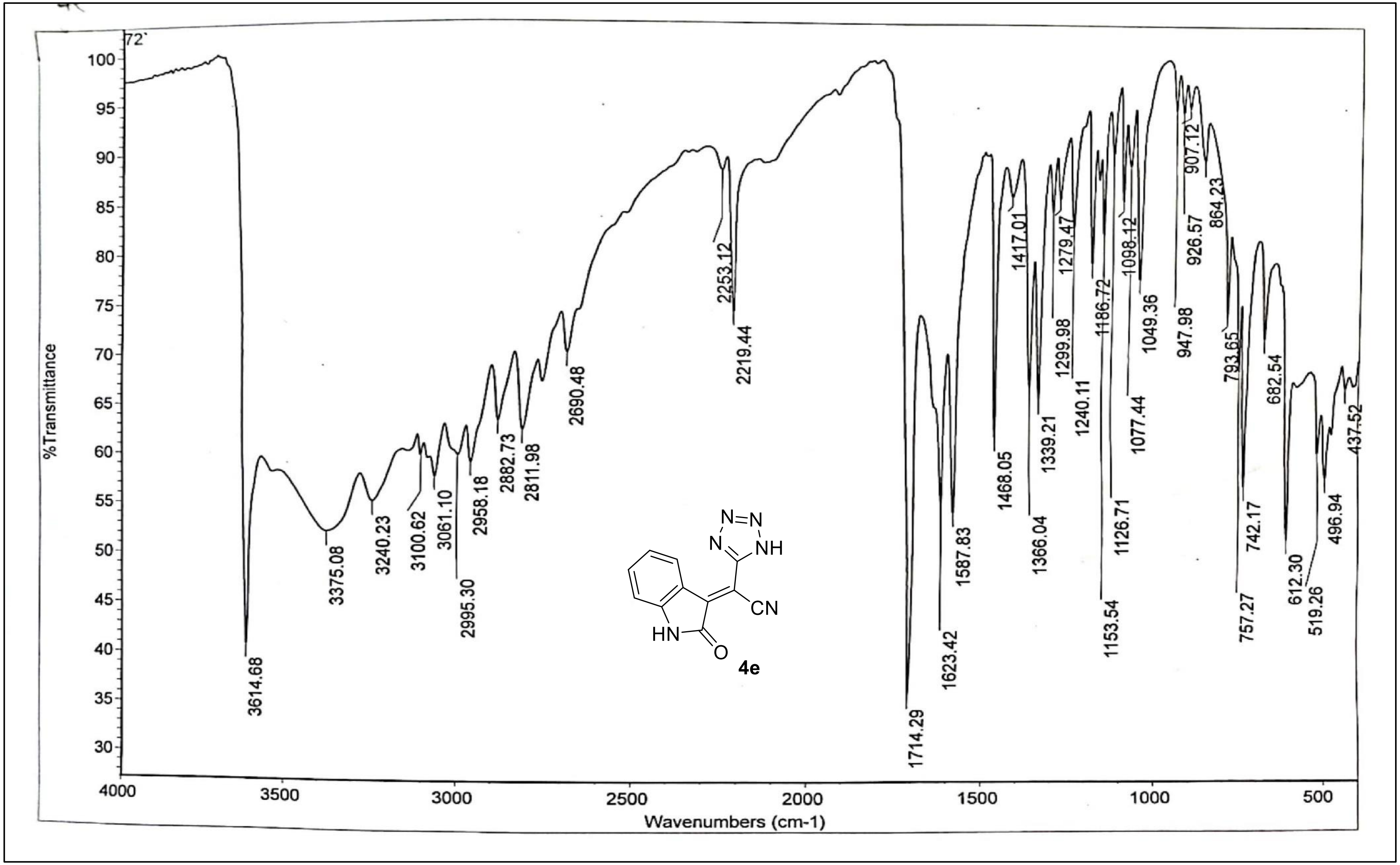

IR spectrum of compound **4e**.

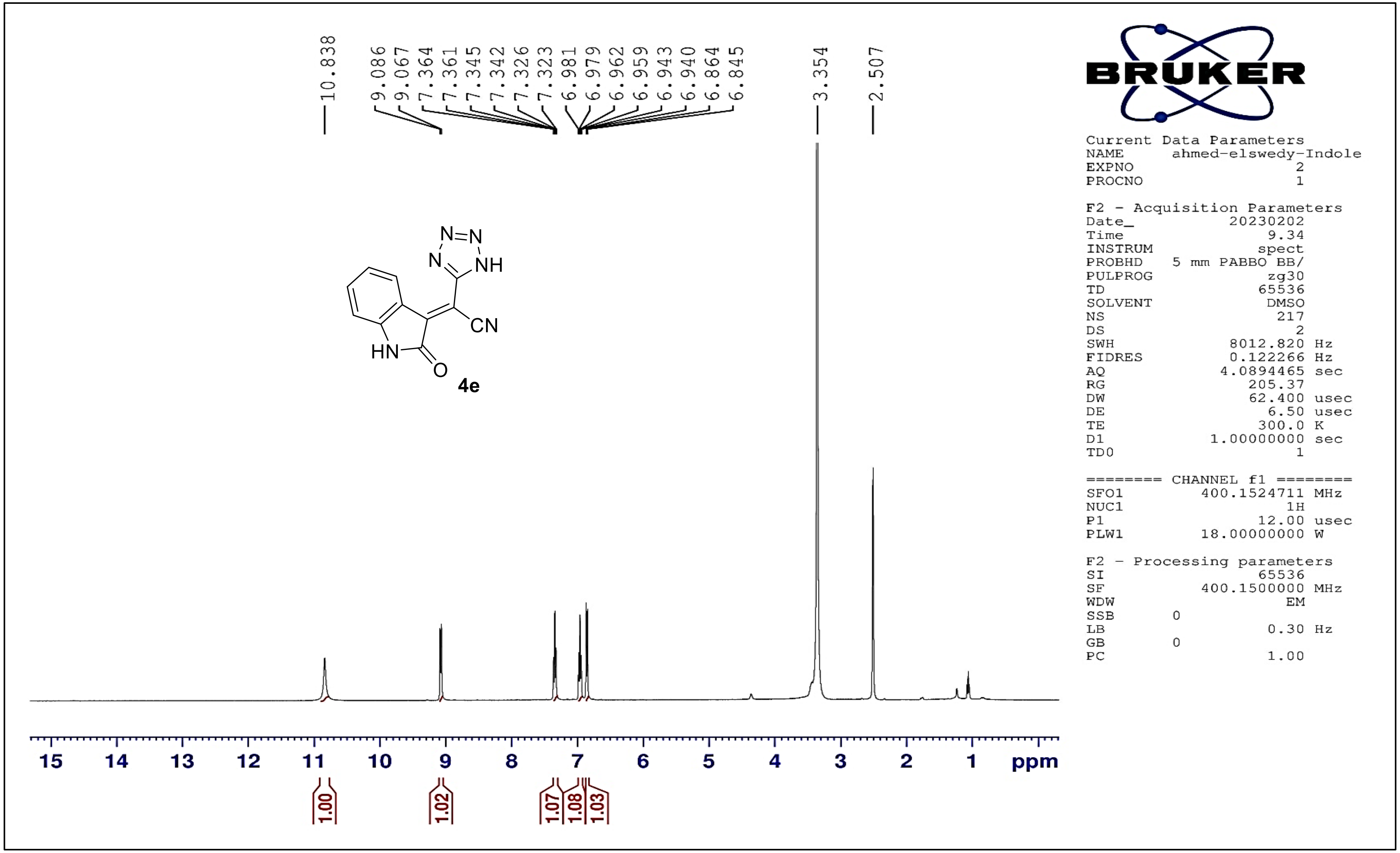

^{1}H NMR spectrum (DMSO-d_6) of compound **4e**.

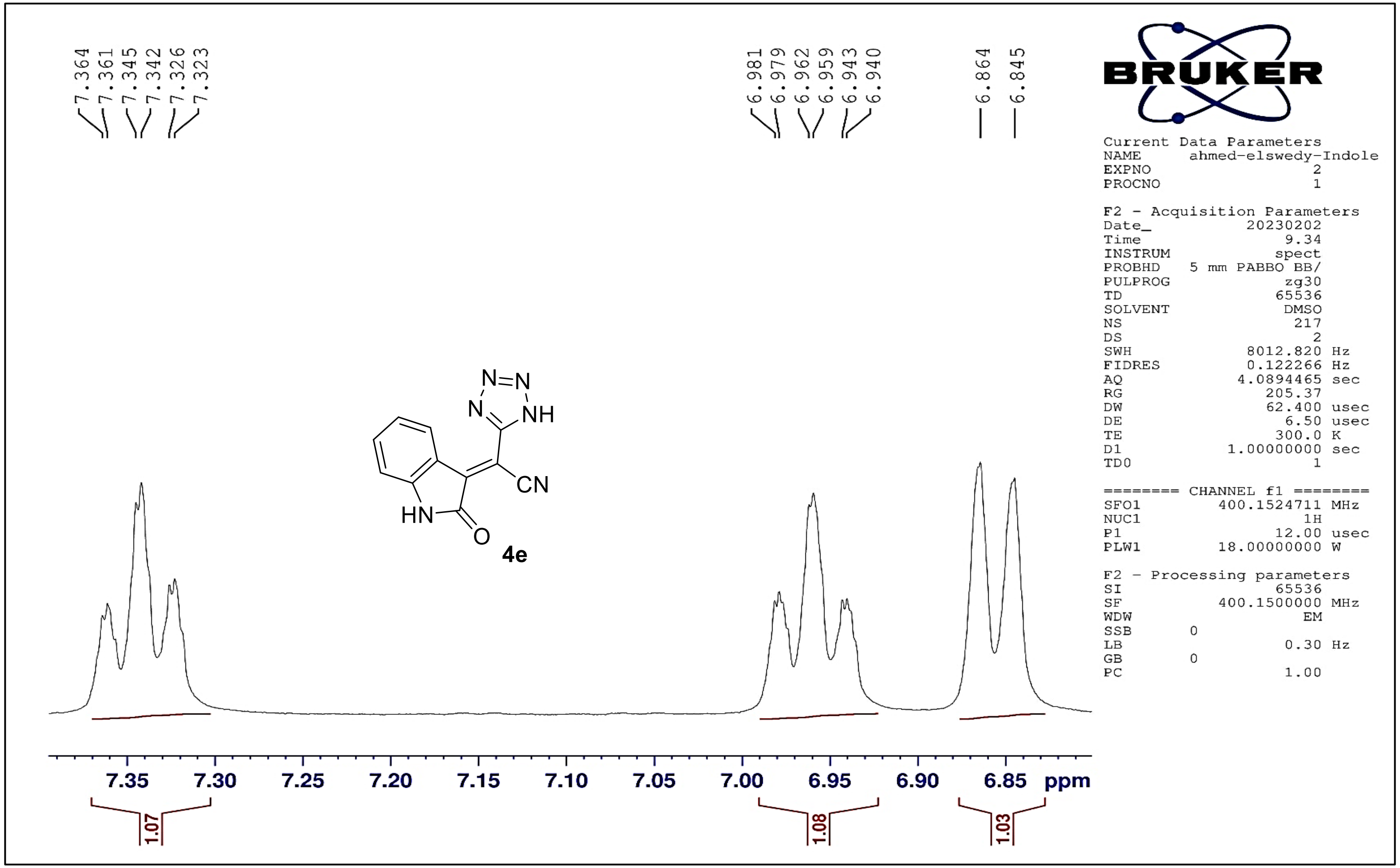

Cont. ^{1}H NMR spectrum (DMSO-d_6) of compound **4e**.

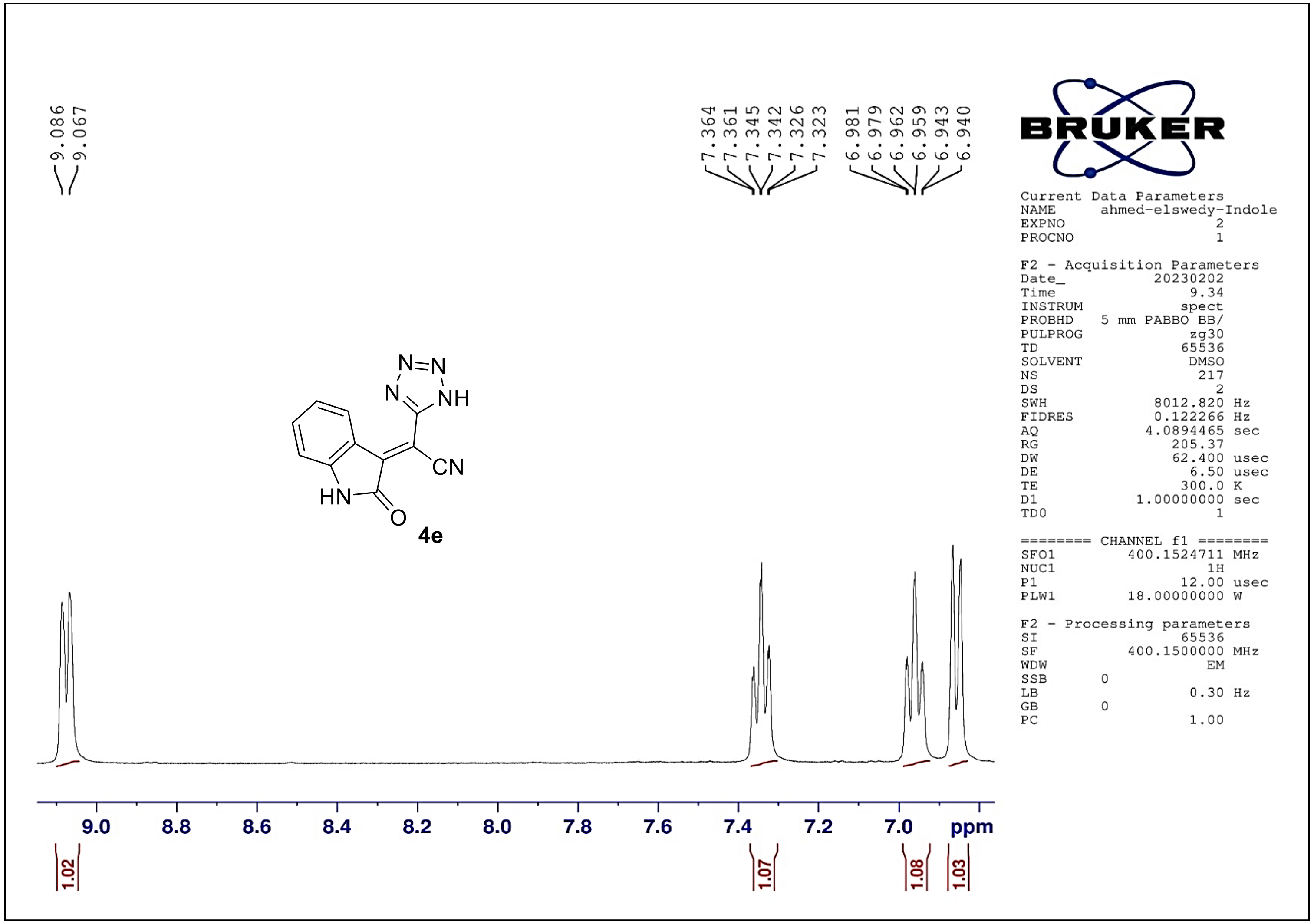

Cont. ^{1}H NMR spectrum (DMSO-d_6) of compound **4e**.

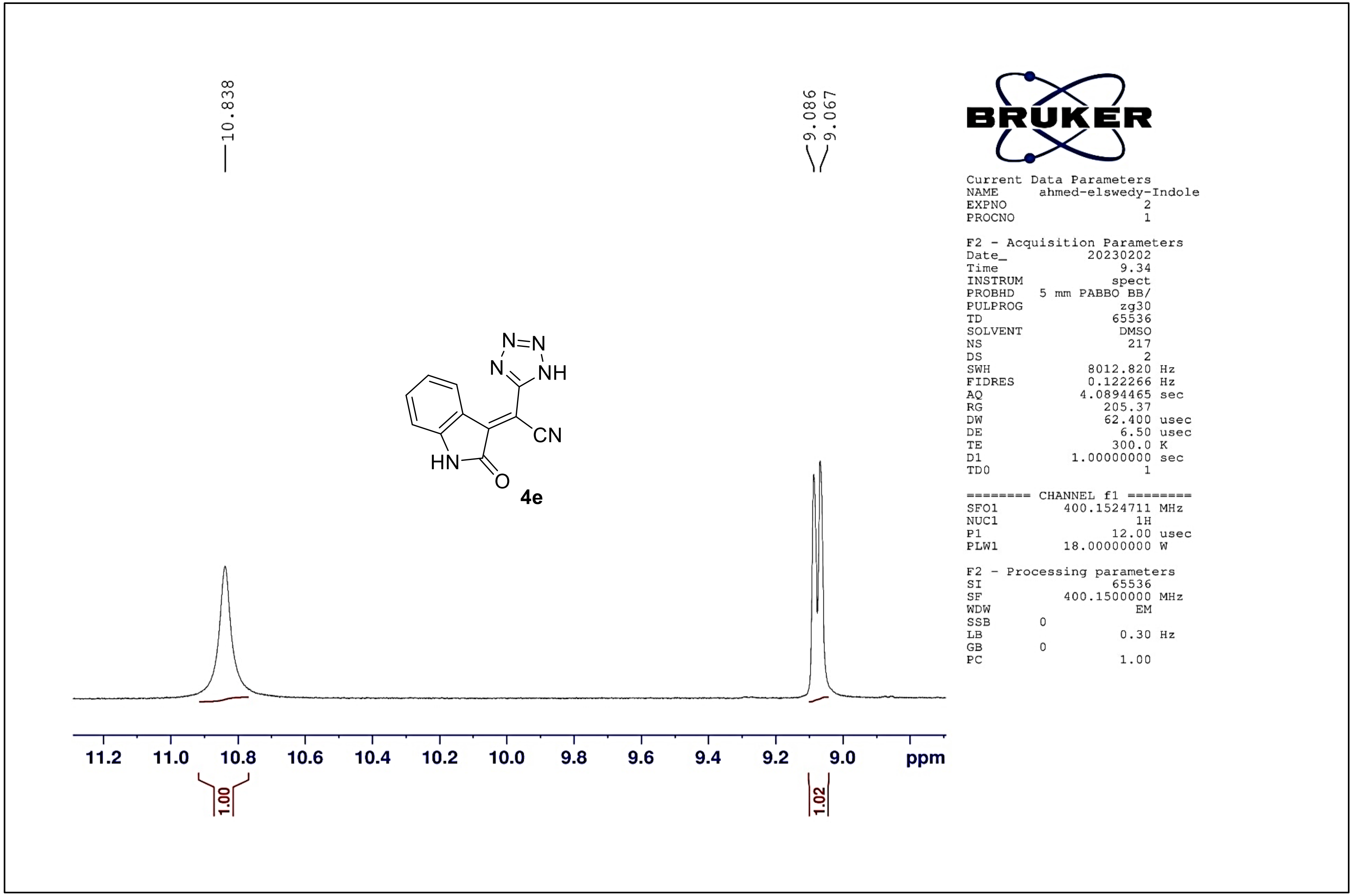

Cont. ^{1}H NMR spectrum (DMSO-d_6) of compound **4e**.

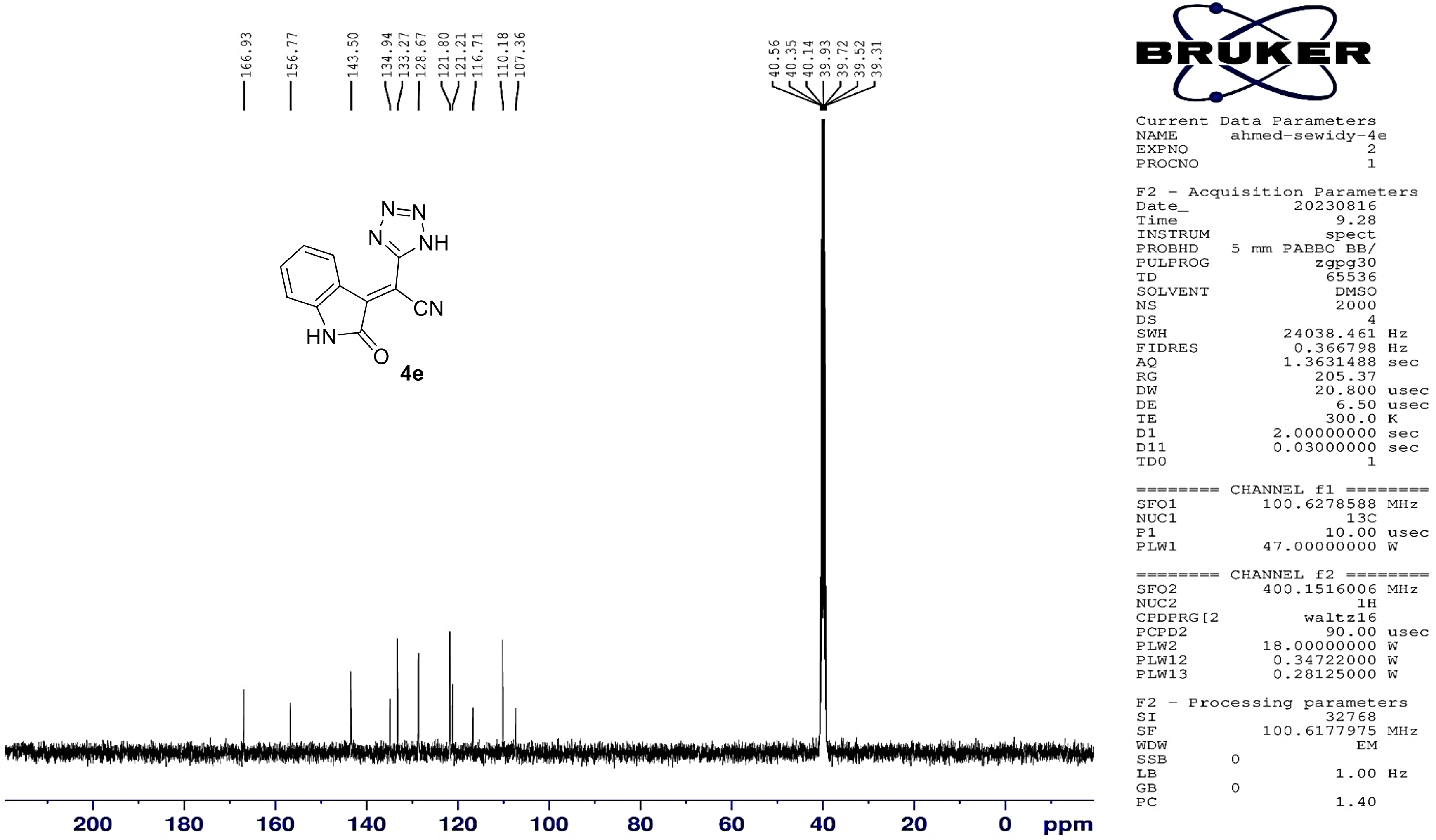

^{13}C NMR spectrum (DMSO-d_6) of compound **4e**.

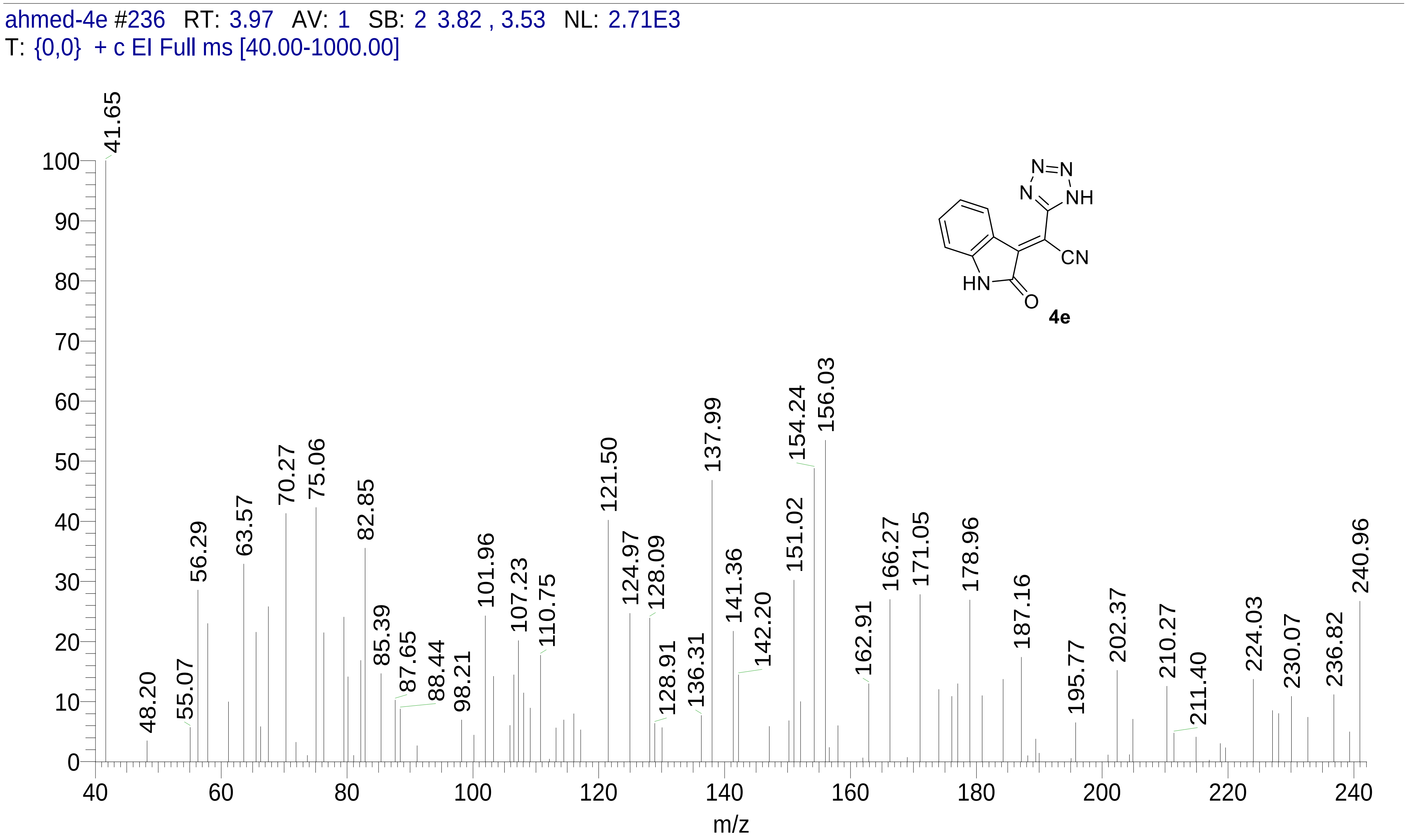

Mass spectrum of compound **4e**.
